# Targeting complement C3 with Tanshinone I decreases microglia-mediated synaptic engulfment to exert antidepressant effects

**DOI:** 10.7150/thno.115587

**Published:** 2025-07-24

**Authors:** Huaqing Lai, Pinglong Fan, Pengxiang Zhang, Meng Zhang, Xinmu Li, Boyu Kuang, Run Zhou, Wenfei Wang, Hong Jiang, Zhenzhen Wang, Naihong Chen

**Affiliations:** 1 School of Pharmaceutical Sciences, Shenzhen Hospital of Integrated Traditional Chinese and Western Medicine, Guangzhou University of Chinese Medicine, Guangzhou 510006, China; 2 State Key Laboratory of Bioactive Substances and Functions of Natural Medicines, Institute of Materia Medica & Neuroscience Center, Chinese Academy of Medical Sciences and Peking Union Medical College, Beijing 100050, China; 3 School of Pharmacy, Hunan University of Chinese Medicine & Hunan Engineering Technology Center of Standardization and Function of Chinese Herbal Decoction Pieces, Changsha 410208, China; 4 College of traditional Chinese medicine and food engineering, Shanxi University of Chinese Medicine, Taiyuan 030024, China.

**Keywords:** Tanshinone I, depression, microglia, synaptic engulfment, fMRI-based network changes

## Abstract

**Background:** The limitations of current depression treatments highlight the importance of developing new therapeutic strategies. Tanshinone I (Tan I), a naturally occurring lipophilic diterpene compound, has promising activities including inflammation inhibition, cellular autophagy or apoptosis modulation, and anti-oxidative stress. However, the potential antidepressant effects of Tan I and the mechanism behind its action have yet to be established.

**Methods:** The antidepressant effect of Tan I was evaluated using animal behavior tests. The chronic unpredictable stress (CUS) mice and C3 overexpressing mice were used to investigate the mechanism of Tan I in microglia-mediated synaptic engulfment, and to explore the effect of Tan I on the improvement of functional magnetic resonance imaging (fMRI)-based network changes in depression-like mice.

**Results:** Here, it is found that Tan I efficiently improved the CUS-induced depressive-like behaviors, attenuated synaptic loss, and inhibited microglial activation. The drug affinity responsive target stability assay and microscale thermophoresis revealed that the specific target of Tan I is complement C3. Furthermore, Tan I decreased the CUS-induced synaptic loss by inhibiting the deposition of C3 deposition onto synapses and subsequent microglia-mediated synaptic engulfment. Importantly, Tan I also improved fMRI-based network changes in CUS mice. Overexpression of C3 in the medial prefrontal cortex (mPFC) of normal mice leads to depressive-like behavior, accompanied by synaptic loss and reduced fMRI-based network changes. In contrast, administration of Tan I inhibits microglia-mediated synaptic phagocytosis and improves fMRI-based network changes, which in turn ameliorate the depressive-like behaviors in C3-overexpressing mice.

**Conclusions:** Collectively, the study demonstrated that Tan I acts as a potent natural C3 modulator that binds directly to C3, blocks the C3-CR3 axis and downstream signal transducer and activator of transcription 3 (STAT3) signaling pathway, inhibits microglia-mediated synaptic engulfment, and improves fMRI-based network changes, which in turn exert antidepressant effects.

## Introduction

The most widespread mental disorder, depression, creates substantial economic challenges for individuals, families, and society 1. The last several decades have seen an evolution in theories concerning the pathophysiological mechanisms of depression. The theory that depression is caused by a decreased level of brain monoamines 2, known as the 'monoamine deficiency hypothesis', was once widely accepted but does not adequately explain the delayed effects and limited efficacy of classical antidepressants. While psychotherapy interventions, as well as chronic antidepressant treatment, have been shown to prevent the onset of psychiatric disorders such as depression 3-5, these interventions still have certain limitations and do not completely satisfy the needs of those with depression. Hence, it is crucial to develop a compound that is convenient, efficient, and safe to curb the progression of depression.

Stress affects neuronal plasticity, which in turn influences the development of psychiatric disorders 6,7. Exposure to stressors can lead to the remodeling of neurons in the prefrontal cortex, which are highly sensitive to stress 8. Chronic stress impacts the dendritic structure of pyramidal neurons in the rodent prefrontal cortex, resulting in reduced dendritic spine density and synaptic plasticity 9,10. Furthermore, it has been shown that social defeat stress mice selectively decrease the neuronal activity in the medial prefrontal cortex 11.

An early and important feature of brain circuit dysfunction in depression is synapse loss 9. There is mounting evidence that microglia-synapse interactions are responsible for synapse loss and dysfunction 12. In recent studies, microglia have been recognized as crucial controllers of synaptic connections in both healthy and diseased brains. Microglia regulate synaptic pruning during development by engulfing and removing some of the less-active synapses 12. A significant mechanism is the phagocytic signaling that relies on the classical complement cascade 13,14. Components of the classical complement cascade, such as C3, in the peripheral immune system, bind to the surfaces of cellular debris and invading pathogens, which are then cleared by professional phagocytes expressing complement receptors 15,16. Microglia that express the C3 receptor, known as CR3, proceed to engulf the synapses associated with complement. Microglia have been shown to mediate synapse engulfment via the complement cascade in neurodegenerative diseases 12. Hence, inhibiting excessive synaptic loss mediated by the complement cascade might serve as a potential method to prevent depression from developing. More importantly, studies have shown that synaptic loss is associated with functional magnetic resonance imaging (fMRI)-based network change abnormalities in depression 17. In adults with major depressive disorder (MDD), fMRI studies have demonstrated disrupted functional connectivity in brain networks 18,19. Therefore, it is crucial to investigate the effects of complement-mediated synaptic loss on fMRI-based network changes in depression.

Due to their remarkable effectiveness and safety, natural products have traditionally been a primary source of drugs aimed at preventing and treating numerous diseases 20. Tanshinone I (Tan I), a naturally occurring lipophilic diterpene compound, has promising activities including inflammation inhibition, cellular autophagy or apoptosis modulation, and anti-oxidative stress 21. Moreover, Tan I has potential applications in areas such as neurodegenerative diseases 22, among others. However, the potential antidepressant effects of Tan I and the mechanism behind its action have yet to be established. Here, it is found that Tan I acts as a potent natural C3 modulator that binds directly to C3, blocks the C3-CR3 axis and downstream signal transducer and activator of transcription 3 (STAT3) signaling pathway, inhibits microglia-mediated synaptic engulfment, and improves fMRI-based network changes, which in turn exert antidepressant effects. These discoveries form a theoretical underpinning for Tan I antidepressant drug research and its clinical implementation.

## Materials and Methods

### Materials

Supplementary Table S1 provides the sources for drugs, antibodies, chemicals, and instruments used in this study.

### Animals

Adult male C57BL/6J mice (7-8 weeks old, 20-22 g) were kept in cages with a 12 h light/dark cycle and had unrestricted access to food and water. The Institutional Animal Care and Use Committee of the Peking Union Medical College and Chinese Academy of Medical Sciences approved all experimental protocols (Ethical inspection No. 00006309), adhering to the National Institutes of Health Guide for the Care and Use of Laboratory Animals.

### Cell culture

Culturing of BV2 and SH-SY5Y neuroblastoma cells was done in DMEM containing 10% FBS at 37 °C in a 5% CO_2_ incubator, and they were passaged every 48 h. From 1-day-old C57BL/6J mice, primary astrocytes were prepared. In short, mice were put under anesthesia, and their brains were swiftly removed. The brains were cut into small pieces using scissors and then incubated in a 0.25% trypsin-EDTA solution at 37 °C for 5 min. The isolated cells were resuspended in DMEM/F12 medium containing 10% FBS and cultured at 37 °C in a 5% CO_2_ incubator. Cultures reached confluence after two weeks, and astrocytes were collected by shaking the flask for 4 h to detach oligodendrocytes and microglia.

### Cell counting kit-8 (CCK-8) assay

In 96-well plates, BV2 cells, SH-SY5Y cells, or primary astrocytes were seeded and given the specified treatment. Following this, the cells underwent a 2 h incubation with the CCK-8 Kit. We then used a microplate reader (Thermofisher, Multiskan GO, CA, USA) to record the absorbance at 450 nm.

### CUS procedure

CUS stress was derived from previous research with certain changes 23,24. Over 8 weeks, mice were exposed to stressors including cage tilting (45°, 24 h), inversion of the light/dark cycle, food deprivation (24 h), damp sawdust (24 h), exposure to an empty cage (24 h), water deprivation (24 h), shaker (180 rpm, 4 h), white noise (4 h), ice-water bath (4 °C, 5 min), tail pinch (5 min), and hot water bath (40 °C, 5 min). To maintain the unpredictability of the experiment, mice were subjected to these stressors in a random sequence.

### Sucrose preference test (SPT)

The SPT was considered a method for assessing anhedonia. The SPT is divided into two phases: adaptation and testing. First was the adaptation phase of SPT, where mice were given sucrose solution (1%) or water for 24 h. At the end of the adaptation period, mice were without food and water for 24 h, after which they were allowed to freely choose between two identical bottles containing either water or a sucrose solution (1%), and the experiment lasted for 4 h.

### Open field test (OFT)

The OFT was employed to reflect behaviors associated with anxiety. During the OFT, mice were positioned in a wooden box (50 cm×50 cm×40 cm), featuring a white bottom that was sectioned into 16 equal parts. The wooden box was wiped with 10% alcohol before experimenting. For 5 min, each mouse was permitted to roam freely within the box, and their activities were tracked and recorded using a high-definition video camera. The data collected were analyzed with Smart version 3.0 software.

### Tail suspension test (TST)

The TST was considered a method to assess behavioral despair. During the TST, the experimental environment was maintained quietly, and the mice were hung upside down and secured with tape at the proper tail suspension position. The cumulative immobile time for each mouse was recorded during the last 4 min of a 6 min period.

### Forced swim test (FST)

The FST was considered a method to assess behavioral despair. Mice were compelled to swim in an open cylindrical container (25 cm in height and 10 cm in diameter), with a water temperature of 23-25 °C during the experiment. Each mouse swam for 6 min, and the cumulative immobile time during the last 4 min was recorded.

### Detection of the brain distribution of Tan I

Tan I (90 mg/kg) was given to mice via intragastric administration, and they were sacrificed at intervals of 15, 30, 60, or 120 min. The brain tissues were then weighed, homogenized in 0.9% NaCl solution, centrifuged, and the supernatant collected. A centrifuge tube was filled with 100 µL of brain sample supernatant, then 300 µL of acetonitrile was added and mixed using a vortex for 2 min. Next, the sample was centrifuged, and the supernatant was pipetted into a new centrifuge tube, where it was concentrated by nitrogen blowing. Following this, 100 µL of acetonitrile was used to re-dissolve the samples and injected into the UltiMate 3000 UPLC system (Shimadzu, Kyoto, Japan) and AB SCIEX 4500 QTRAP mass spectrometer (SCIEX, MA, USA) for analysis. The mobile phase included acetonitrile (A) and 0.1% formic acid in water (B), using a gradient program of 0-1 min, 10%A; 1-8 min, 10%-90%A; and 8-10 min, 90%-10%A, and the transitions monitored for quantification were *m/z* 277.0 > 178.0 for Tan I.

### Golgi staining

After perfusion, the brains were collected from mice and promptly placed into the Golgi dye solution (Servicebio, Beijing, China). For 14 days, the process was conducted in the dark (after 48 h of soaking, the dye solution was replaced with a new one, and then with a new one every 3 days for a total of 14 days). The tissue was dehydrated with 30% sucrose after dyeing and then cut into sections of 100 µm.

### Transmission electron microscopy

The medial prefrontal cortex (mPFC) in mice brain tissues was placed in 2.5% glutaraldehyde for 2 h at 4 °C and then fixed with 1% osmium tetroxide for 2 h. After dehydration with cold-graded ethanol, and then embedded in resin. Then, the above tissues were sliced into 90 nm-thick ultrathin sections, and the images were obtained with an electron microscope (JEOL, Tokyo, Japan).

### Immunofluorescence

Following continuous perfusion with 4% paraformaldehyde, the brains of the mice were isolated and fixed in a 4% paraformaldehyde solution. After dehydration in a gradient of sucrose, the brains were cut into slices with a thickness of 30 μm. First, the sections were micro-boiled in citrate antigen repair solution for 10 min. Then, they were treated with 0.5% Triton-X-100, followed by sealing with 5% BSA. Finally, they were incubated overnight at 4 °C with target protein antibodies. After incubation with the primary antibody, incubate with the corresponding immunofluorescent secondary antibody for 2 h at room temperature. Sections were imaged with a Cytation C10 (Agilent BioTek, CA, USA) confocal imaging reader.

### Gene expression analysis

First, the mPFC of mice brains was taken, and then total RNA was extracted with TRIzol reagent (Invitrogen, CA, USA). Reverse transcription was used to prepare the cDNA, and the 7500 Real-time PCR system (Applied Biosystems, CA, USA) was employed to measure the relative expression of mRNAs. Supplementary Table S2 provides the sequences of primers employed for real-time PCR.

### RNA sequencing

First, the mPFC of mice brains was taken, and then total RNA was extracted with a TRIzol reagent. Then, reverse transcriptase was used to reverse transcribe the RNA fragments from the Veh + CUS and Tan I + CUS groups to generate cDNA. Finally, sequencing (PE150) was performed on an Illumina Novaseq 6000 (Illumina, CA, USA). High-quality data was obtained by filtering the off-machine data from high-throughput sequencing. Genes that differed between sample groups were screened, and visualized displays like volcanic diagrams and clustering analysis were created for these differential genes.

### Western blot analysis

First, total protein was extracted from the mPFC of mice brains using RIPA lysis buffer. The proteins were then quantified using the BCA protein assay kit. Then, gel electrophoresis was performed with 10% SDS-PAGE, followed by transferring the proteins onto 0.45 μm PVDF membranes. The PVDF membranes were blocked with 5% BSA, and then the bands were incubated with the primary antibodies corresponding to the target proteins at 4 °C overnight. The next day, the bands were incubated with the corresponding secondary antibodies at room temperature for 2 h. Using Image Quant LAS 4000 (GE, Boston, USA), the protein bands were visually captured, and Image J software was employed to evaluate their comprehensive density.

### Stereotaxic injection of lentivirus

C3 Lentiviral (LV) was injected bilaterally into the PFC at these coordinates: AP, +0.19 mm; ML, ±0.03 mm; and DV, -0.21 mm. The viral constructs used in this study included pcSLenti-CMV-EGFP-3×FLAG-WPRE (LV-NC, titer 5.39×10^8^ TU/mL), pcSLenti-CMV-C3-3×FLAG-WPRE (LV-C3, titer 3.69×10^8^ TU/mL), and pcSLenti-CMV-C3 (K1084G, L1088F, Q1277G, Q1281A)-3×FLAG-WPRE (LV-C3mut, titer 5.53×10^8^ TU/mL). These LVs were obtained from OBiO Technology (Shanghai, China). Briefly, a Hamilton syringe was used to inject 1.0 μL of the LV solution over 10 min. The syringe was kept in place for an extra 10 min after injection to allow adequate viral diffusion.

### MRI data acquisition and processing

The PharmaScan 70/16 (Bruker BioSpin, Ettlingen, Germany) was used to generate MRI datasets, with image acquisition performed by ParaVision_6.0.1 software (Bruker BioSpin GmbH). To ensure stable positioning, animals were first anesthetized with a continuous flow of 2% isoflurane in a 70/30% N_2_/O_2_ gas mixture. At the start of the scan, the isoflurane concentration was lowered to 1.5%. To enhance the uniformity of the magnetic field at the start of every MRI exam, a FieldMap and continuous local gaskets are employed, followed by T2-weighted TurboRARE sequences for anatomical reference scans. Functional MRI is then acquired using a free induction decay echo-planar imaging (FID-EPI) sequence, comprising 300 repetitions with a field of view (FOV) of 20 × 20 mm², an echo time (TE) of 15 ms, and a repetition time (TR) of 2000 ms. We conducted Pearson correlation analyses between ALLF/ReHo values of brain regions with significant differences and the behavioral test index to examine the relationship between brain changes and assessed behaviors induced by Tan I. A p-value of less than 0.05 was considered statistically significant.

The extraction of brain functional data sets was conducted by converting the original data into nifti format files using dcm2nii (version 2MAY2016). The voxel size was magnified tenfold, with the middle layer serving as the reference for temporal layer correction. Once cephalometric correction was completed, all the subjects' data were registered to the standard space with the ants toolkit (version 1.9.2), utilizing a two-step registration approach. Use T2 and the average function image to register the function image to the Allen Mouse Brain 25. Subsequently, we utilize the restplus V1.2.8-130615 and SPM12 toolkits to execute filtering and Gaussian smoothing on the registered data. In the end, following de-linearization and regression covariate analysis, we acquire the data prepared for analysis.

The functional connectivity of the brain region was assessed by calculating the correlation coefficients among the seed points. Statistical analysis was conducted using MATLAB R2013b, and BrainNetViewer (v1.61) was employed to visualize the functional connectivity results 26.

### Molecular dynamics (MD)

First, the structure of the complement C3 protein was obtained from the PDB database, followed by the structure of Tan I from the PubChem database. Docking between Tan I and C3 was performed using Auto Dock Vina, and finally, PyMOL (v2.2.0) visualized the docking results. The simulations of molecular dynamics were initiated from small molecule-protein complexes acquired by docking, and these were carried out using the AMBER 22 software.

### Microscale thermophoresis (MST)

RED-NHS was used to label the C3 proteins. After replacing the buffer, purifying the protein, and balancing, a 0.12 μM solution of labeled protein was achieved. The Monolith NT.115 instrument was used for all measurements at 25 °C. Tan I compounds were dissolved in PBS with 5% DMSO to make stock solutions. The binding affinities of Tan I and C3 were determined using Monolith NT.115 (NanoTemper Technologies, Munich, Germany).

### Hematoxylin and eosin (H&E) staining

In short, the slices were heated at 60 °C for 2 h, then deparaffinized and rehydrated through a series of ethanol concentrations until reaching deionized water. The slices were then stained with hematoxylin for 10 min and eosin for 2 min, followed by dehydration in graded ethanol and clearing in xylene.

### Mass spectrometry (MS)-based drug affinity responsive target stability (DARTS) assay

The DARTS assay is used to identify proteins that bind to small-molecule drugs, based on the principle that these drugs can decrease the protease sensitivity of the proteins they bind to. BV2 cell proteins, ranging from 50 to 100 μg, were incubated with either 200 μM Tan I or DMSO for 1 h. Subsequently, the proteins underwent digestion with varying Pronase protease concentrations for 30 min. Finally, the proteins obtained by digestion with protease were separated through SDS-PAGE gel electrophoresis. The levels of the targeting protein were assessed using Coomassie brilliant blue or Western blotting. Moreover, mass spectrometry analysis using the Q Exactive HF-X (Thermo Fisher Scientific, MA, USA) was conducted to identify the proteins based on the differential bands observed from Coomassie brilliant blue staining.

### Cellular thermal shift assay (CETSA)

The principle of CETSA is that small-molecule drugs increase the thermal stability of the proteins to which they bind. Before digestion by proteases, the procedure mirrors that of the DARTS assay. Different temperatures (40, 45, 50, 55, 60, and 65 °C) were used to degrade each protein part for 3 min, but not the proteases. Following this, all samples were centrifuged. Finally, the content of target proteins in the supernatant was detected by Western blotting assay.

### Statistical analysis

The Shapiro-Wilk test was used to check the data for normality, and SPSS 29.0 was employed to assess statistical significance. Student's t test was used to analyze differences between two groups, while one-way ANOVA with Tukey's post hoc test was used for multiple comparisons.* P* < 0.05 was considered statistically significant. All the statistical analyses were performed using GraphPad Prism 9.0 software (GraphPad, CA, USA). The data are expressed as mean ± SEM.

## Results

### Tan I improves the CUS-induced depressive-like behaviors in mice

To determine whether Tan I has the potential for antidepressant effects, we first explored this by establishing an in *vitro* depression cell model. Our results showed that in three common in *vitro* cell models of depression, corticosterone (CORT)-induced SH-SY5Y cells, CORT-induced primary astrocytes, and LPS-induced BV2 cells, the survival rate of the cells was significantly enhanced by Tan I treatment (*F*_(5, 30)_ = 46.19, *P* < 0.001, Figure S1A; *F*_(5, 30)_ = 12.32, *P* < 0.001, Figure S1B; *F*_(5, 30)_ = 13.90, *P* < 0.001, Figure S1C), suggesting that Tan I has excellent antidepressant potential.

The TST and FST are widely used in antidepressant screening 27. To initially assess the antidepressant effects of Tan I, behavioral tests were conducted on all mice without any stress exposure (Figure S1D). Our results showed that after 2 weeks of Tan I administration, the immobilization time of TST and FST was significantly reduced in mice with Tan I doses of 30 and 90 mg/kg compared with vehicle, whereas there was no significant difference with 10 mg/kg (*F*_ (4, 35)_ = 5.982, *P* < 0.001, Figure S1E; *F*_(4, 35)_ = 6.268, *P* < 0.001, Figure S1F). Fluoxetine (Flu) (10 mg/kg) significantly shortened the immobility time of TST and FST. The results of TST and FST suggest that Tan I may have antidepressant potential. In addition, there were no significant differences between Tan I (10, 30, or 90 mg/kg) or Flu (10 mg/kg) in OFT in terms of the total distance, the distance in the center, the time spent in the center, and the number of entries in the center (*F*_(4, 35)_ = 0.09947, *P* = 0.9819, Figure S1G; *F*_(4, 35)_ = 0.08414, *P* = 0.9868, Figure S1H; *F*_(4, 35)_ = 0.4739, *P* = 0.7546, Figure S1I; *F*_(4, 35)_ = 0.1870,* P* = 0.9436, Figure S1J). Figure S1K displays the representative animal traces of mice from various groups in the OFT. This suggests that the effects of Tan I on TST and FST are not related to spontaneous activity in mice. The above results indicated that Tan I and Flu significantly decreased the immobility time of mice in TST and FST, suggesting that Tan I has antidepressant effects, with effective doses being 30 mg/kg and 90 mg/kg.

**Figure 1:**
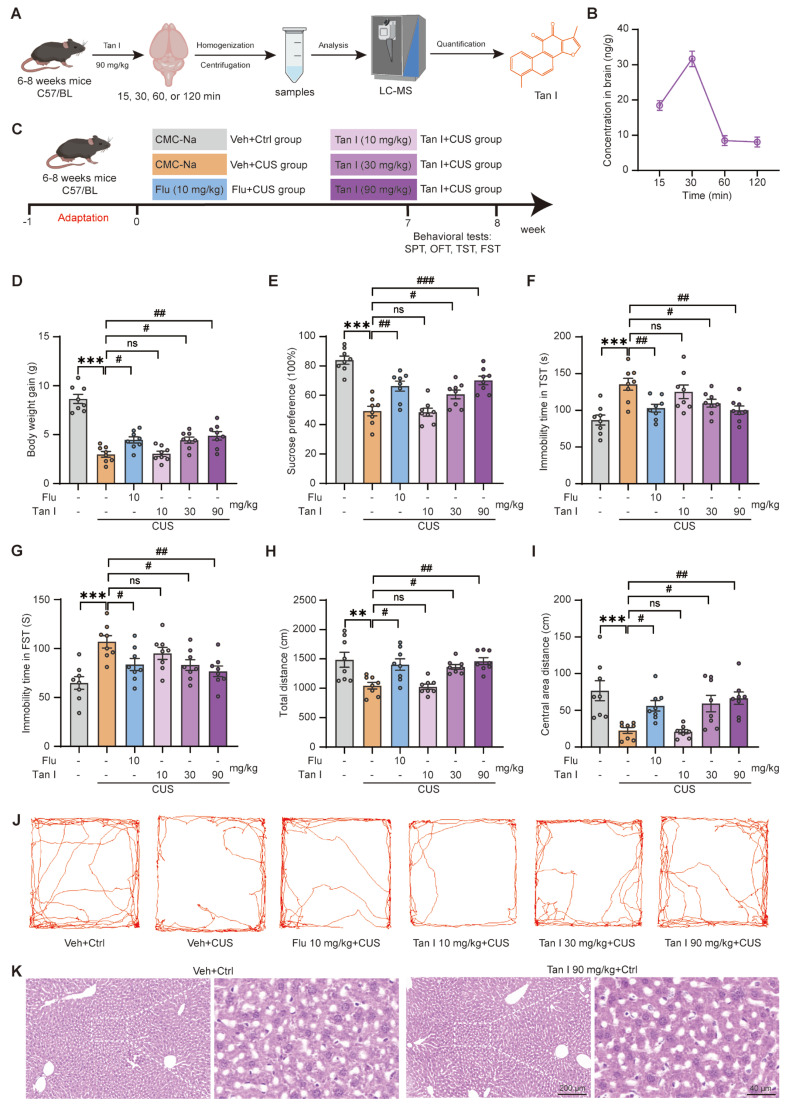
** Tan I attenuates the CUS-induced depressive-like behaviors. (A)** Schematic diagrams for the determination of Tan I content. **(B)** The concentration curve of Tan I in the brain tissues of mice at different time points (15, 30, 60, and 120 min) after Tan I administration (n = 3). **(C)** The mice were split into six groups, and behavioral tests were conducted in the eighth week. **(D)** The mice administered Tan I at doses of 30 and 90 mg/kg showed a significant increase in body weight compared to the CUS mice (n = 8). **(E)** Tan I (30 or 90 mg/kg) significantly improved the SPT test in CUS mice, while Tan I (10 mg/kg) did not affect the SPT test compared to the CUS group (n = 8). **(F-G)** Tan I (30 or 90 mg/kg) significantly decreased the immobility time in the TST (**F**) and FST (**G**), while Tan I (10 mg/kg) did not affect either the TST or FST compared to the CUS group (n = 8). **(H-I)** Tan I (30 or 90 mg/kg) significantly increased the total distance **(H)** and the distance in the center **(I)** of the OFT, while Tan I (10 mg/kg) did not affect the OFT compared with the CUS group (n = 8). **(J)** Representative traces of mice's movement in the OFT. **(K)** HE staining images of liver tissue sections from vehicle control or Tan I control groups. Scale bar = 200 μm (left), 40 μm (right). ^∗∗^*P* < 0.01, ^∗∗∗^*P* < 0.001 versus vehicle control group; ^#^*P* < 0.05, ^##^*P* < 0.01,^ ###^*P* < 0.001 versus vehicle CUS group; ns, not significant. One-way ANOVA with Tukey's post hoc test. All data are expressed as mean ± SEM.

To evaluate whether Tan I, a natural lipophilic diterpene compound, can cross the blood-brain barrier (BBB) in mice, its brain levels were measured with LC-MS/MS following an administration dose of 90 mg/kg (Figure 1A). Our findings indicate that Tan I reached a peak concentration of 30 ng/g in the brain (Figure 1B), implying its ability to cross the BBB in mice.

To further assess if Tan I could exhibit anti-depression effects, mice were administered vehicle (0.5% CMC-Na) or 10, 30, and 90 mg/kg doses of Tan I, respectively. Subsequently, the mice were exposed to CUS and administered respective doses of Tan I over 8 weeks (Figure 1C). Interestingly, we studied the body weights of the mice and showed a significant increase in body weight in the Tan I group at 30 and 90 mg/kg doses compared to the CUS group (*F*_(5, 42)_ = 34.31, *P* < 0.001, Figure 1D). Furthermore, our study showed that administering 30 and 90 mg/kg of Tan I significantly improved the SPT in CUS mice, while a 10 mg/kg dose of Tan I did not affect the SPT compared to the CUS group (*F*_(5, 42)_ = 19.85, *P* < 0.001, Figure 1E). In addition, Tan I (30 or 90 mg/kg) significantly decreased the immobility time in the TST (*F*_(5, 42)_ = 6.529, *P* < 0.001, Figure 1F) and FST (*F*_(5, 42)_ = 5.899, *P* < 0.001, Figure 1G), while Tan I (10 mg/kg) did not affect either the TST or FST compared to the CUS group. Similarly, Tan I (30 or 90 mg/kg) significantly increased the total distance (*F*_(5, 42)_ = 7.166, *P* < 0.001, Figure 1H) and the distance in the center (*F*_(5, 42)_ = 7.088, *P* < 0.001, Figure 1I) of the OFT, while Tan I (10 mg/kg) did not affect the OFT compared with the CUS group. Figure 1J displays the representative animal traces of mice from various groups in the OFT. Therefore, our results demonstrated that administering 30 and 90 mg/kg of Tan I significantly improved depression-like behaviors in the SPT, TST, FST, and OFT tests in CUS mice, while a 10 mg/kg dose of Tan I had no effects on these behaviors compared to the CUS group.

Furthermore, we conducted HE staining on liver tissues from mice that received a continuous 8-week administration of Tan I at 90 mg/kg, and the findings indicated no significant pathological damage in the liver tissues (Figure 1K). We demonstrated that administering 30 and 90 mg/kg of Tan I or 10 mg/kg of Flu significantly improved depression-like behaviors in CUS mice, with 90 mg/kg of Tan I being the most effective. Consequently, 90 mg/kg of Tan I was opted for in subsequent in *vivo* studies.

### Tan I improves the neuronal structure and neuronal activity in the mPFC of CUS mice

**Figure 2:**
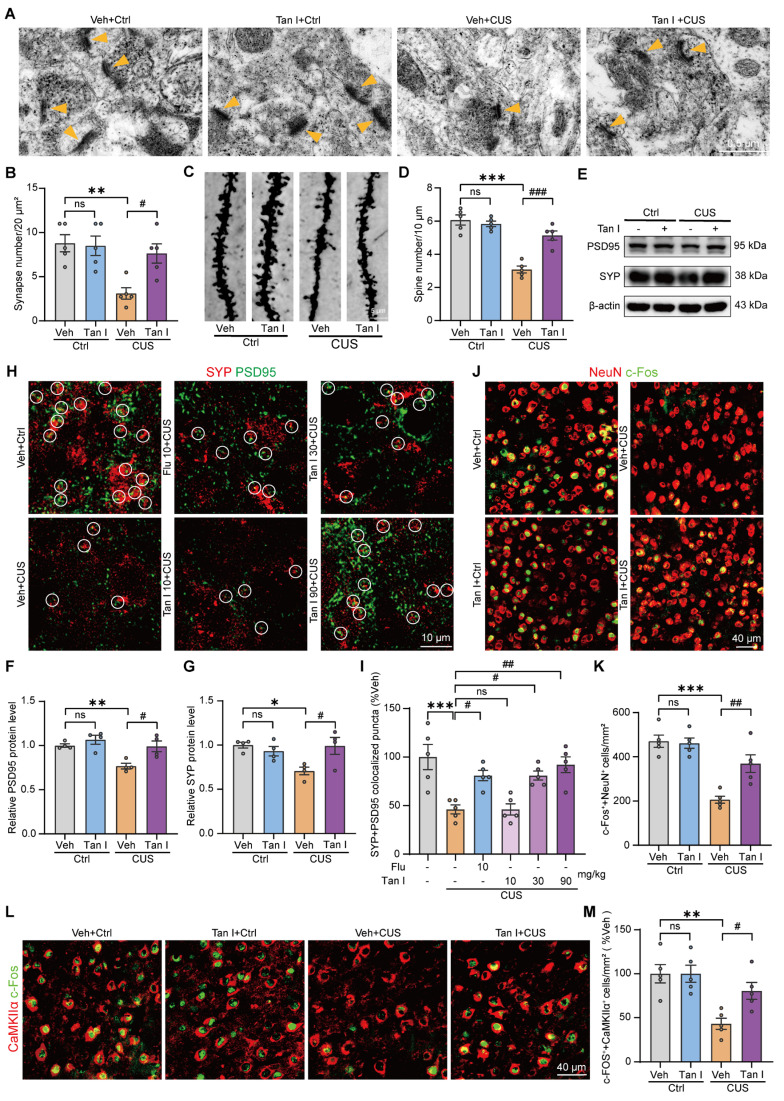
** Tan I improves the neuronal structure and neuronal activity in the mPFC of CUS mice. (A)** The representative transmission electron microscopy of different groups of mice (n = 5). Scale bar = 0.5 µm. Orange arrows show synapses. **(B)** The number of synapses per 20 μm^2^ was calculated (n = 5). **(C)** Golgi-Cox staining shows representative images of the dendritic spines of pyramidal neurons from different groups of mice. Scale bar = 5 μm.** (D)** Quantification of dendritic spine density of pyramidal neurons in different groups of mice (n = 5). **(E)** The levels of PSD95 and SYP in the mPFC were analyzed. **(F-G)** Relative quantification of PSD95 and SYP protein levels of CUS mice after Tan I treatment (n = 4). **(H)** Representative images of PSD95 (green) and SYP (red) in different groups of mice. Scale bar = 10 μm. Colocalized puncta were indicated by circles. **(I)** Quantification of the colocalization of SYP and PSD95 in different groups of mice (n = 5). **(J-K)** Representative images **(J)** and quantitative analysis **(K)** of NeuN^+^ (red) and c-Fos^+^ (green) in the mPFC of CUS mice after Tan I treatment (n = 5). Scale bar = 40 μm. **(L-M)** Representative images **(L)** and quantitative analysis **(M)** of c-Fos^+^ (green) and CaMKIIα^+^ (red) in the mPFC of CUS mice after Tan I treatment (n = 5). Scale bar = 40 μm. ^∗^*P* < 0.05, ^∗∗^*P* < 0.01, ^∗∗∗^*P* < 0.001 versus vehicle control group; ^#^*P* < 0.05, ^##^*P* < 0.01,^ ###^*P* < 0.001 versus vehicle CUS group; ns, not significant. One-way ANOVA with Tukey's post hoc test. All data are expressed as mean ± SEM.

It has been shown that neurons in the mPFC area are extremely sensitive to stress 10. We utilized transmission electron microscopy to assess whether Tan I has protective effects on the neuron structure by examining synapse characteristics and numbers in the mPFC of CUS mice. Our study revealed that in CUS mice, there was a reduction in the number of synapses (*F*_(3, 16)_ = 7.407, *P* = 0.0025, Figure 2B), as well as the thickness (*F*_(3, 76)_ = 5.708, *P* = 0.0014, Figure S2A) and length (*F*_(3, 76)_ = 22.75, *P* < 0.001, Figure S2B) of postsynaptic density in the mPFC, while the cleft width of synapses was significantly increased (*F*_(3, 76)_ = 3.871, *P* = 0.0124, Figure S2C). However, these impairments were significantly improved after Tan I treatment compared to the CUS group (Figure 2A-B and Figure S2A-C). Additionally, Golgi Staining was used to analyze spine density changes. The Golgi Staining analysis revealed that CUS mice exhibited a significant decrease in dendritic spine density in the mPFC relative to the control, whereas Tan I treatment significantly increased the dendritic spine density (*F*_(3, 16)_ = 31.22, *P* < 0.001, Figure 2C-D).

Research indicates that synapse loss is a key characteristic of psychiatric disorders caused by stress 9, so we investigated whether it exists in the mPFC of CUS mice. We conducted a Western blot analysis to determine if Tan I treatment could affect postsynaptic density 95 (PSD95) and synaptophysin (SYP) levels in the mPFC of CUS mice. We found that Tan I significantly increased PSD95 (*F*_(3, 12)_ = 8.743, *P* = 0.0024) and SYP (*F*_(3, 12)_ = 4.977, *P* = 0.0180) levels compared to the CUS group (Figure 2E-G). Further, through immunofluorescence experiments, we observed that PSD95 (postsynaptic marker) and SYP (presynaptic marker) were colocalized (Figure 2H) and noted a significant loss of synapses in the mPFC of CUS mice. However, compared with the CUS group, Tan I at doses of 30 mg/kg and 90 mg/kg both improved synaptic loss in CUS mice, whereas the dose of 10 mg/kg failed to improve synaptic loss. In addition, Tan I at 30 mg/kg was comparable to Flu at 10 mg/kg in improving synaptic loss in CUS mice, while Tan I showed more significant effects at 90 mg/kg (*F*_(5, 24)_ = 9.429, *P* < 0.001, Figure 2I). The above results showed that Tan I improved synaptic loss in CUS mice in a dose-dependent manner.

It is commonly understood that the mPFC region is closely associated with the regulation of maladaptive emotional states, such as depression 8. Hence, we investigated neuronal activity in the mPFC using c-Fos immunostaining after the FST in CUS mice. Our results showed that c-Fos^+^ neurons (colocalized with NeuN) were significantly reduced in CUS mice after FST compared to the control group, while Tan I significantly increased the number of c-Fos^+^ neurons (*F*_(3, 16)_ = 19.11, *P* < 0.001, Figure 2J-K). Additionally, to determine which type of neuron was primarily activated following FST, GAD67 (a marker for inhibitory neurons) or CaMKIIα (a marker for excitatory neurons) was co-stained with the c-Fos antibody for immunofluorescence analysis, respectively, in the control group. Interestingly, the results demonstrated that c-Fos was chiefly colocalized with CaMKIIα, with only a small degree of colocalization with GAD67 (*P* < 0.001, Figure S3A-B). In addition, we analyzed the count of c-Fos^+^ excitatory neurons (colocalized with CaMKIIα) of CUS mice following FST by immunofluorescence. Data from immunofluorescence showed that c-Fos^+^ excitatory neurons were significantly reduced in the mPFC of CUS mice, while Tan I significantly increased the number of c-Fos^+^ excitatory neurons (*F*_(3, 16)_ = 8.575, *P* = 0.0013, Figure 2L-M). The above data indicate that the activity of excitatory neurons was significantly reduced in the mPFC of CUS mice, while Tan I effectively increased the activity of these neurons.

### Tan I inhibits the microglia activation in the mPFC of CUS mice

**Figure 3:**
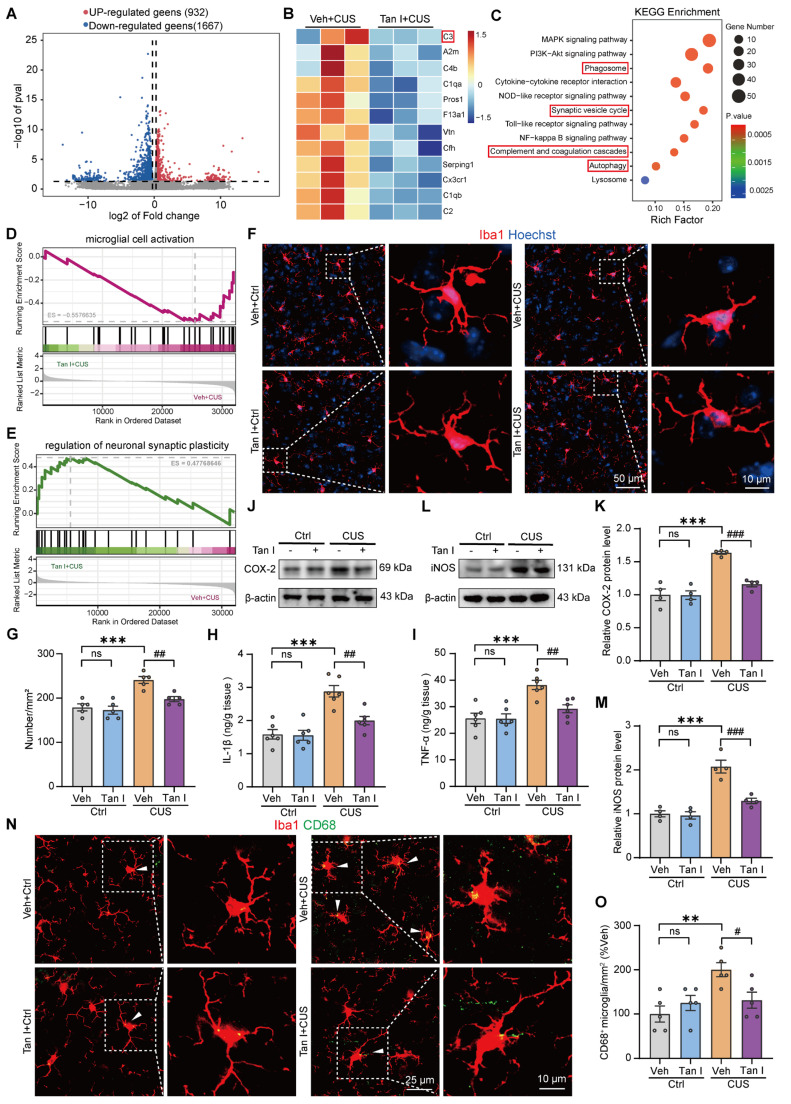
** Tan I inhibits the microglia activation in the mPFC of CUS mice. (A)** The volcano plot showed differentiated genes, with 932 up-regulated genes and 1667 down-regulated genes. **(B)** Heat maps of the DEGs between the Veh + CUS and Tan I + CUS groups were determined by RNA-seq. **(C)** KEGG pathway analysis was performed based on the obtained DEGs. **(D-E)** GSEA analyses revealed that Tan I affected gene expression related to microglial cell activation **(D)** and regulation of neuronal synaptic plasticity **(E)**. **(F-G)** Representative images **(F)** and quantitative analysis **(G)** of Iba1 (red) and Hoechst (blue) in the mPFC of CUS mice after Tan I treatment (n = 5).** (H-I)** The levels of IL-1β (**H**) and TNF-α (**I**) were determined in the mPFC tissues in different groups of mice (n = 4). **(J)** The levels of COX-2 in the mPFC of different groups of mice were analyzed.** (K)** Relative quantification of COX-2 protein levels in different groups of mice (n = 4). **(L)** The levels of iNOS in the mPFC of different groups of mice were analyzed.** (M)** Relative quantification of iNOS protein levels in different groups of mice (n = 4). **(N)** Representative images of Iba1 (red) and CD68 (green) in the mPFC of CUS mice after Tan I treatment (n = 5). Scale bar = 25 μm. Zoom in images (bar = 10 μm). **(O)** The quantification of the number of CD68^+^ microglia (n = 5). ^∗∗^*P* < 0.01, ^∗∗∗^*P* < 0.001 versus vehicle control group; ^#^*P* < 0.05, ^##^*P* < 0.01, ^###^*P* < 0.001 versus vehicle CUS group; ns, not significant. One-way ANOVA with Tukey's post hoc test. All data are expressed as mean ± SEM.

Given that Tan I has an antidepressant effect, we collected the mPFC from mice in the Veh + CUS group and the Tan I + CUS group and investigated the differentially expressed genes (DEGs) using RNA sequencing. The experimental results showed that Tan I treatment altered the gene expression profile. Following Tan I intervention, 932 genes were upregulated in expression, while 1667 genes were downregulated, compared to the CUS group (Figure 3A). Specifically, the expression of complement cascade-related genes, such as C1qa, C1qb, C3, and Cx3cr1, was predominantly reduced after Tan I treatment compared to the CUS group (Figure 3B). Further, we validated these DEGs using quantitative PCR (qPCR). The qPCR results showed similar differential expression patterns to those identified in the RNA-seq data (*P* = 0.0447, Figure S4A; *P* = 0.0122, Figure S4B;* P* < 0.001, Figure S4C;* P* = 0.0042, Figure S4D). Further, the Kyoto Encyclopedia of Genes and Genomes (KEGG) pathway enrichment analysis of DEGs revealed predominantly altered expression of genes linked to the phagosome, synaptic vesicle cycle, complement, and coagulation cascades, and autophagy (Figure 3C). Gene ontology (GO) analysis of DEGs revealed enrichment in pathways, such as complement activation, complement component C1q complex binding, and complement-mediated synapse pruning (Figure S4E). In addition, we conducted gene set enrichment analyses (GSEAs) and discovered that Tan I affected gene expression related to microglial cell activation, regulation of neuronal synaptic plasticity (Figure 3D-E), and synapse assembly (Figure S4F). These findings imply that Tan I could influence microglia-mediated engulfment.

Research has shown that stress can activate microglia, which induce the release of neuroinflammatory cytokines, phagocytosis, and morphological changes 28,29. Further, we detected the number of microglia of CUS mice using an antibody to Iba1 (microglia marker) and thus assessed the effect of Tan I on microglia activation. The results of immunofluorescence revealed that the number of Iba1^+^ microglia in the mPFC of CUS mice was significantly increased, and Tan I inhibited this increase (*F*_(3, 16)_ = 15.79, *P* < 0.001, Figure 3F-G). Furthermore, we examined the levels of neuroinflammation-related cytokines, such as IL-1β, TNF-α, IL-10, TGF-β, COX-2, and iNOS, which are linked to the activation of microglia 30,31. The results indicated that IL-1β and TNF-α levels were significantly elevated in the mPFC of CUS mice compared to controls, but these increases were notably inhibited by Tan I treatment (*F*_(3, 20)_ = 17.50, *P* < 0.001, Figure 3H; *F*_ (3, 20)_ = 11.70, *P* < 0.001, Figure 3I). In addition, IL-10 and TGF-β levels were significantly reduced in the mPFC of CUS mice compared to controls, but were significantly elevated after Tan I treatment (*F*_(3, 20)_ = 5.924, *P* = 0.0046, Figure S5A; *F*_(3, 20)_ = 8.172, *P* = 0.001, Figure S5B). Similarly, Western blot analysis indicated that COX-2 and iNOS levels were significantly higher in the mPFC of CUS mice. However, Tan I reduced the increase in COX-2 and iNOS levels in the mPFC of CUS mice (*F*_(3, 12)_ = 23.62, *P* < 0.001, Figure 3J-K; *F*_(3, 12)_ = 28.79, *P* < 0.001, Figure 3L-M). The above results suggest that Tan I inhibits neuroinflammation in CUS mice. CD68 is known to be a lysosomal marker, indicating the phagocytic activity of microglia. Therefore, we assessed the count of Iba1^+^ (red) and CD68^+^ (green) colocalized in the mPFC of CUS mice to analyze CUS-induced microglia phagocytic activity. Our results showed a significant increase in the number of Iba1^+^ and CD68^+^ colocalization in the mPFC of CUS mice compared to the control group, which was reduced by Tan I (*F*_(3, 16)_ = 6.054, *P* = 0.0059, Figure 3N-O), suggesting that Tan I reduces the phagocytic activity of microglia in CUS mice.

### Tan I interacts with C3 as its specific target

**Figure 4:**
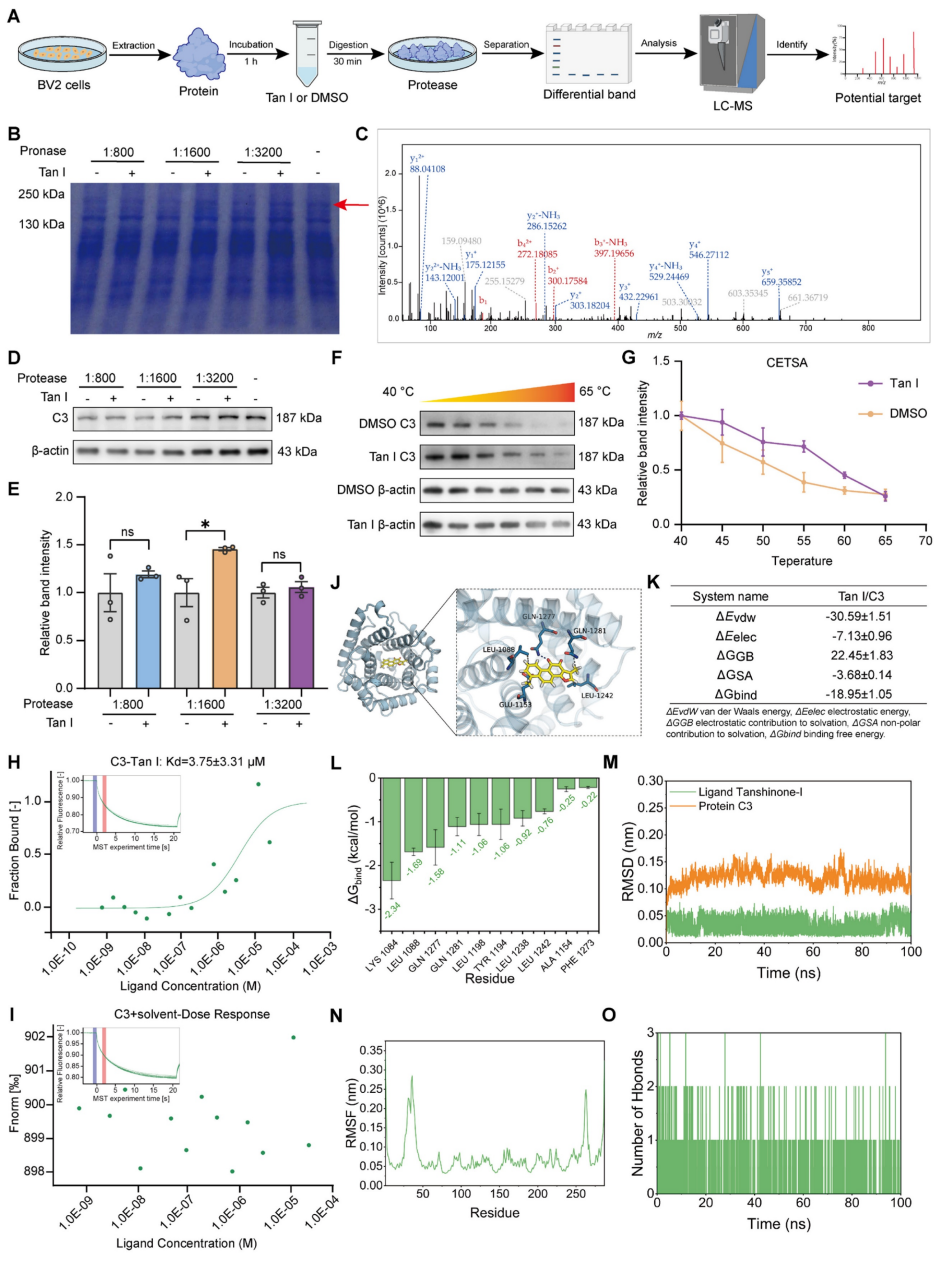
** Tan I interacts with C3 as its specific target. (A)** Schematic diagrams of the DARTS assay. **(B)** In the DARTS assay, Coomassie blue staining of the SDS-PAGE gel showed that Tan I protected the band. **(C)**The panel is the C3 adapted image from mass spectrometry. **(D)** The C3 level was determined by Western blot after conducting the DARTS assay on Tan I/BV2 cells. **(E)** Relative quantification of C3 protein levels was performed (n = 3). **(F)** During the CETSA assay, the proteins from BV2 cells incubated with DMSO or Tan I for 1 h were subjected to various temperatures for denaturation. Western blotting was used to assess the protein level of C3. **(G)** The densitometry analysis curve of F. **(H-I)** MST was used to analyze the binding of fluorescently labeled C3 to Tan I **(H)**, but the solvent control experiment could not be fitted to the binding curve **(I)**. **(J)** The visualized image of molecular docking between C3 and Tan I. **(K)** MM/GBSA calculations derived from MD simulations revealed that the binding free energy (ΔG_bind_) of Tan I to the protein C3 was -18.95 ± 1.05 kcal/mol. **(L)** The MM-GBSA energy catabolism approach was employed to obtain the top 10 amino acids that contribute to the binding of Tan I and C3. **(M)** RMSD results of the complex in the simulation process. **(N)** RMSF results of residues during 50 ns MD. **(O)** The number of hydrogen bonds between Tan I and C3 during MD simulations. ^∗^*P* < 0.05; ns, not significant. Two-tailed Student's t test. All data are expressed as mean ± SEM.

Further, we performed a DARTS assay 32 to screen and identify the specific targets of Tan I. In this assay, BV2 cellular proteins were incubated with Tan I and Pronase protease, and Tan I protected the target proteins from protease degradation (Figure 4A). A distinct band in the 130-250 kDa molecular weight range was observed through SDS-PAGE separation, showing significant differences between DMSO and Tan I (Figure 4B). Next, we performed mass spectrometry analysis on this differential band obtained to detect possible proteins, and based on both theoretical and actual molecular weight sizes, identified C3 as the only protein around 130-250 kDa (Figure 4C), suggesting it as a potential target of Tan I. We conducted a Western blot on DARTS samples to further confirm this discovery, showing that Tan I protected C3 from proteolytic degradation (Figure 4D-E).

CETSA utilizes small-molecule drugs that bind to target proteins to reduce their degradation or thermal denaturation 33, therefore, we also conducted CETSA to further confirm the interaction between Tan I and C3. As the temperature increased, Tan I slowed down the degradation of C3 (Figure 4F). The thermal denaturation curve of the protein moved to the right after Tan I was added (Figure 4G). The above experimental data show that Tan I significantly enhances the thermal stability of C3 in a certain temperature range, confirming the specific interaction between Tan I and C3. MST was applied to assess if Tan I directly interacts with C3. A combined binding curve was generated by incubating C3 with different concentrations of Tan I, with the maximum concentration being 25 µM. The Kd value was determined to be 3.75 ± 3.31 µM (Figure 4H). Furthermore, the solvent control experiments showed that the binding curves could not be fitted (Figure 4I). In addition, we used MST to investigate whether there were also interactions between Tan I and other complement components, such as C1q and C4. The MST results showed that Tan I did not interact with either C1q (Figure S6A) or C4 (Figure S6B). All these findings suggest a direct interaction between Tan I and C3, and that C3 is a Tan I-specific target.

We conducted molecular docking analysis to examine the binding affinities and interactions between Tan I and C3. Tan I has a binding affinity score of -7.153 kcal/mol with C3, indicating a relatively strong interaction with the C3 protein (Figure 4J). To further uncover the interaction and binding site of Tan I with C3, an MD simulation of the Tan I-C3 complex was employed. MM/GBSA calculations derived from MD simulations revealed that the binding free energy (ΔG_bind_) of Tan I to the protein C3 was -18.95 ± 1.05 kcal/mol (Figure 4K). The MM-GBSA energy catabolism method was employed to obtain the amino acids that are the top contributors to the binding of Tan I and C3, specifically LYS 1084, LEU 1088, GLN 1277, and GLN 1281. These amino acids are thus critical for Tan I and C3 binding (Figure 4L). The interaction between Tan I and C3 was stable according to MD simulation, and Tan I binding led to a more compact structure of C3 molecules, and the formation of many hydrogen bonds with small molecules (Figure 4M-O).

All the above results show that Tan I and C3 can directly bind to their specific target, and the binding site may be LYS 1084, LEU 1088, GLN 1277, and GLN 1281 amino acids.

### Tan I inhibits microglia-mediated synaptic engulfment of CUS mice to exert antidepressant effects

**Figure 5:**
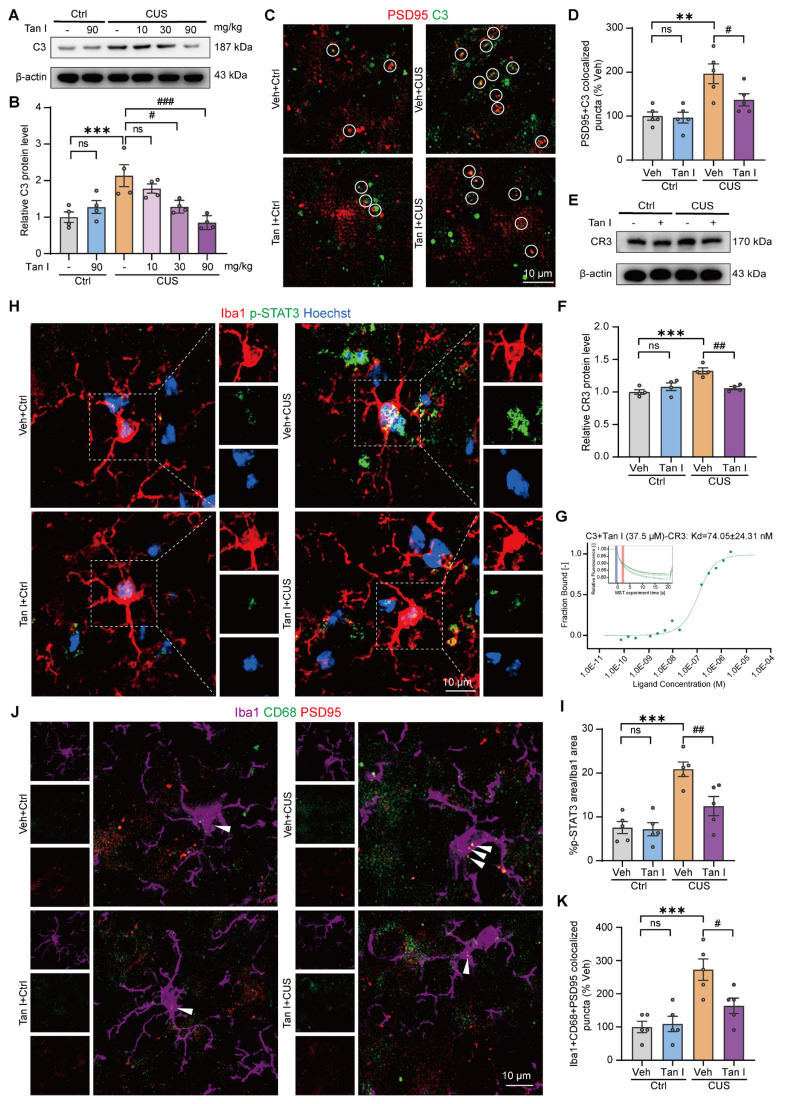
** Tan I inhibits microglia-mediated synaptic engulfment of CUS mice to exert antidepressant effects. (A)** The levels of C3 in the mPFC of CUS mice after Tan I treatment were analyzed. **(B)** Relative quantification of C3 protein levels of CUS mice after Tan I treatment (n = 4). **(C)** Representative images of PSD95 (green) and C3 (red) in the mPFC. Scale bar = 10 μm. Colocalized puncta were indicated by circles. **(D)** Quantification of the colocalization of PSD95 and C3 in different groups of mice (n = 5). **(E)** The levels of CR3 in the mPFC were analyzed. **(F)** Relative quantification of CR3 protein levels of CUS mice after Tan I treatment (n = 4). **(G)** MST was used to determine the incubation of different concentrations of CR3 with C3-Tan I complexes to form binding curves, and the Kd value was calculated as 74.05 ± 24.31 nM. **(H)** Representative images of Iba1 (red) and p-STAT3 (green) in different groups of mice. Scale bar = 25 μm. **(I)** The quantification of the ratio of the p-STAT3 area to the Iba1 area in different groups of mice (n = 5). **(J)** Representative images of Iba1^+^ microglia (purple) containing PSD95^+^ puncta (red) and CD68^+^ (green) in the mPFC. White arrowheads showed the colocalization between PSD95 and CD68 in microglia. Scale bar = 10 μm. **(K)** Quantification of the colocalization of PSD95^+^ and CD68^+^ in Iba1^+^ microglia in different groups of mice (n = 5). ^∗∗^*P* < 0.01,^ ∗∗∗^*P* < 0.001 versus vehicle control group;^ #^*P* < 0.05, ^##^*P* < 0.01, ^###^*P* < 0.001 versus vehicle CUS group; ns, not significant. One-way ANOVA with Tukey's post hoc test. All data are expressed as mean ± SEM.

In the brain, the complement system is crucial for regulating microglial synaptic pruning. It has been observed that complement molecules, including C1q and C3, are located at synapses and mediate synapse loss in neurodegenerative diseases through the action of phagocytic microglia 12,34,35. Complement C3 induces the engulfment of labeled synapses by microglia by promoting the growth of chemotactic microglia and binding to their CR3 receptor. We hypothesize that C3 could also be localized at the synapse following CUS. For this purpose, we investigated C3 expression in the mPFC of CUS mice. Interestingly, consistent with the behavioral results, we demonstrated that 30 and 90 mg/kg Tan I significantly reduced the increase in C3 in the mPFC of CUS mice, and that 90 mg/kg was more effective, while 10 mg/kg Tan I did not affect C3 levels compared to the CUS group (*F*_(5, 18)_ = 8.115, *P* < 0.001, Figure 5A-B). These results show that the inhibition of C3 by Tan I is dose-dependent. Subsequently, we examined the colocalization of C3 and PSD95 in CUS mice. Our immunofluorescence images revealed a greater percentage of PSD95^+^ colocalized with C3 in the mPFC of CUS mice. The colocalization of PSD95^+^ with C3 was significantly reduced by Tan I, implying that Tan I reduces C3 deposition at synapses in CUS mice (*F*_(3, 16)_ = 9.230, *P* < 0.001, Figure 5C-D).

Since C3 is linked to synaptic engulfment by microglia, we examined the expression of CR3, which is a receptor specific to C3 in microglia 36. We investigated the expression of CR3 in the mPFC and found that the expression level of CR3 was significantly elevated in CUS mice compared to controls. However, Tan I reduced the CR3 increase in the mPFC of CUS mice (*F*_(3, 12)_ = 10.86, *P* = 0.001, Figure 5E-F). The activation of the C3-CR3 axis has been shown to play an essential role in microglia-mediated phagocytosis of synapses. Complement C3 induces microglia to engulf labeled synapses by binding to CR3 receptors on chemotactic microglia 37. Therefore, we investigated whether Tan I blocks the interaction between C3 and its receptor, CR3, through MST assays. Incubated with different concentrations of CR3 (maximum concentration of 2.9 μM) and fluorescently labeled C3, a combined binding curve was generated, and the Kd value was determined to be 5.73 ± 10.8 nM (Figure S7A). The complex was formed by the addition of 37.5 μM Tan I to C3, followed by incubation with different concentrations of CR3 and the C3-Tan I complex, resulting in a binding curve with a Kd value calculated to be 74.05 ± 24.31 nM (Figure 5G). In addition, the MST results showed that there was no interaction between CR3 and Tan I (Figure S7B). The above results indicate that Tan I and CR3 competitively bind to C3, and Tan I could block the interaction between C3 and its receptor, CR3. Further, we assessed the count of Iba1^+^ (red) and CR3^+^ (green) colocalized in the mPFC. Our results showed a significant increase in the number of Iba1^+^ and CR3^+^ colocalization of CUS mice compared to the control group, which was reduced by Tan I (*F*_(3, 16)_ = 9.167, *P* < 0.001, Figure S8A-B).

The STAT3 signaling pathway is an important downstream signaling pathway activated by C3, and inhibition of the STAT3 signaling pathway can alleviate neuroinflammation 38. When STAT3 is activated in microglia, it promotes their transformation into reactive microglia, which then perform several functions, such as synaptic pruning 39. To explore the potential downstream pathways through which Tan I inhibits the effects of C3, we investigated the STAT3 signaling pathway. Interestingly, the level of p-STAT3 expression was significantly higher in the mPFC of CUS mice, while Tan I significantly downregulated the level of p-STAT3 in the mPFC of CUS mice (*F*_(3, 12)_ = 6.106, *P* = 0.0092; *F*_(3, 12)_ = 7.911, *P* = 0.0035; Figure S9A-C). Furthermore, we performed immunofluorescence experiments, which showed that p-STAT3 expression was significantly upregulated in microglia in the mPFC of CUS mice, while Tan I inhibited this increase compared to the CUS group (F _(3, 16)_ = 14.15, *P* < 0.001, Figure 5H-I). These results suggest that Tan I inhibits C3 and affects the downstream STAT3 signaling pathway. Additionally, we examined the co-localization of PSD95 and CD68 in microglia in the mPFC. Our findings indicated that the proportion of PSD95 colocalized with CD68 was significantly higher in microglia from CUS mice. Nonetheless, Tan I markedly decreased these colocalizations compared to the Veh + CUS group (*F*_(3, 16)_ = 10.52, *P* < 0.001, Figure 5J-K). These findings imply that Tan I lessens synapse loss in CUS mice by inhibiting C3 deposition on synapses and microglia-induced synaptic engulfment.

### Tan I improves fMRI-based network changes in CUS mice

**Figure 6:**
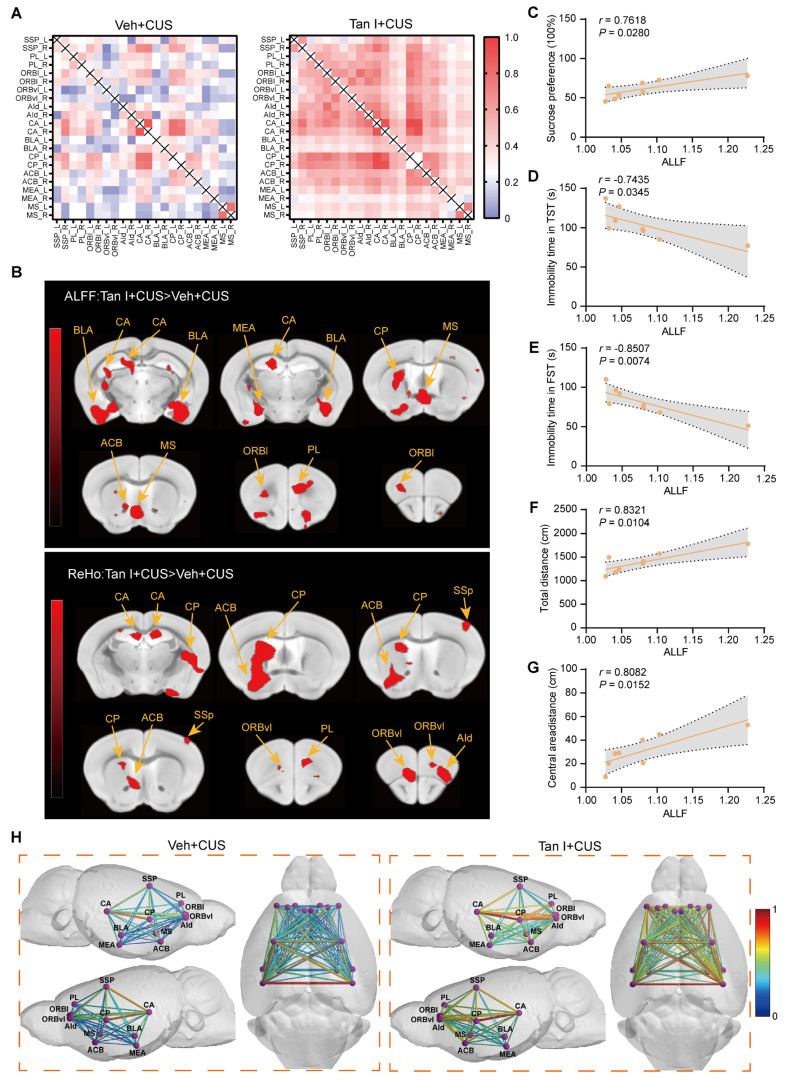
Tan I improves fMRI-based network changes in CUS mice. **(A)** The results of the heatmap showed that the Z-score matrices were lower in the Veh + CUS group of mice, whereas they increased after treatment with Tan I. **(B)** Comparative analysis of the ALFF and Reho values between the Tan I + CUS group and the Veh + CUS group. Different colors represent brain regions with significant differences based on Z-score values. **(C-G)** The correlation analysis between the behaviors with alterations in ALFF in PL. **(H)** The functional correlations between different brain regions are represented by lines, with redder colors and thicker lines indicating higher Z-score values. N = 4 mice.

Studies have shown that synaptic loss is associated with fMRI-based network change abnormalities in depression 17. Consequently, we analyzed the differences in functional connectivity between the Tan I + CUS and Veh + CUS groups in mice. We conducted brain scans on CUS mice treated with Tan I and collected fMRI data. We conducted a region-specific analysis of fMRI data focusing on areas mainly impacted by depression 40, selecting regions of interest (ROIs) such as the primary somatosensory area (SSp), prelimbic area (PL), orbital area, lateral part (ORBl), orbital area, ventrolateral part (ORBvI), agranular insular area (AId), field CA (CA), basolateral amygdalar nucleus (BLA), caudoputamen (CP), nucleus accumbens (ACB), medial amygdalar nucleus (MEA) and medial septal nucleus (MS), for a total of 11 brain regions 40. MATLAB tools were used to process the fMRI data 41. The Z-score values were used to obtain the functional connectivity matrices for the Veh + CUS and Tan I + CUS groups, with redder colors indicating stronger connectivity between regions. The Tan I + CUS group displayed a generally redder functional connectivity matrix compared to the Veh + CUS group, suggesting that Tan I treatment can improve fMRI-based network changes in CUS mice (Figure 6A).

In addition, we analyzed abnormal low-frequency fluctuation (ALFF) and regional homogeneity (ReHO) in the Veh + CUS group and the Tan I + CUS group to evaluate the overall effect of Tan I on brain activity in CUS mice. ALFF indicates the amplitude of spontaneous neuronal activity in certain areas 41. The voxel-level threshold for ALFF, without correction, was established at *P* < 0.01, with a cluster extent threshold of 20 voxels. Our results showed that ALFF levels were increased in the Tan I + CUS group compared to the Veh + CUS group, mainly in regions including the PL, ORBl, CA, BLA, CP, ACB, MEA, and MS (Figure 6B). ReHO was used to indicate the consistency of neuronal activity in regions 41. For the ReHO analysis, the voxel-level threshold was also set to *P* <0.01, with a cluster range threshold of 10 voxels. We found that Reho levels were elevated in the Tan I + CUS group compared to the Veh + CUS group, mainly in brain regions including the SSp, PL, ORBvI, AId, CA, CP, and ACB (Figure 6B).

The PL is especially sensitive to depression and is a key region influenced by the mPFC. Therefore, we focused on alterations in the connectivity strength between PL and other regions associated with depression. The relationship between CUS mice behavior (SPT, OFT, TST, and FST) and ALFF values of PL was explored by correlation analysis. According to the study results, there was a notable correlation between the behavioral indices and the ALFF value of PL in CUS mice (Figure 6C-G). Notably, the ALFF value of PL had a negative correlation with the immobilization time in both the TST and FST in CUS mice (Figure 6D-E). The findings offer further neuroimaging evidence that Tan I improves depressive-like behaviors.

Further, we visualized the data using lines to show the connections between specific brain regions, with redder colors and thicker lines signifying stronger connectivity. In the Veh + CUS group, the lines appeared bluer and thinner, but following Tan I treatment, they became redder and thicker (Figure 6H), suggesting improved connectivity. All of the above findings indicate that Tan I treatment improves fMRI-based network changes between brain regions in CUS mice.

### Tan I inhibits microglia-mediated synaptic engulfment of C3 OE mice to exert antidepressant effects

Given that Tan I inhibits C3 deposition on synapses and microglia-mediated synapse engulfment to exert antidepressant effects, we injected lentivirus-mediated C3 overexpression bilaterally into the PFC of WT mice to mimic the pathology of elevated C3 (Figure S10A). The results of PCR (*F*_(3, 12)_ = 5.901, *P* = 0.0103, Figure S10B) and Western blot (*F*_(3, 12)_ = 17.75, *P* < 0.001, Figure S10C-D) showed that the levels of C3 were significantly elevated in the mPFC of C3 OE mice compared with C3 NC mice. Whereas Tan I treatment significantly reduced the C3 levels compared to the C3 OE mice.

Notably, C3 OE mice exhibited depressive-like behaviors. Our study showed that administering 90 mg/kg of Tan I significantly improved depression-like behaviors in the SPT, TST, FST, and OFT in C3 OE mice (*F*_(3, 28)_ = 9.709, *P* < 0.001, Figure S10E; *F*_(3, 28)_ = 14.03, *P* < 0.001, Figure S10F; *F*_(3, 28)_ = 16.42, *P* < 0.001, Figure S10G; *F*_(3, 28)_ = 6.397, *P* = 0.0019; *F*_(3, 28)_ = 4.969, *P* = 0.0069, Figure S10H-J), indicating that the anti-depression effect of Tan I might be mediated by inhibiting the expression of C3. The above results suggest that Tan I exerts anti-depressant effects in the mPFC by inhibiting C3.

**Figure 7:**
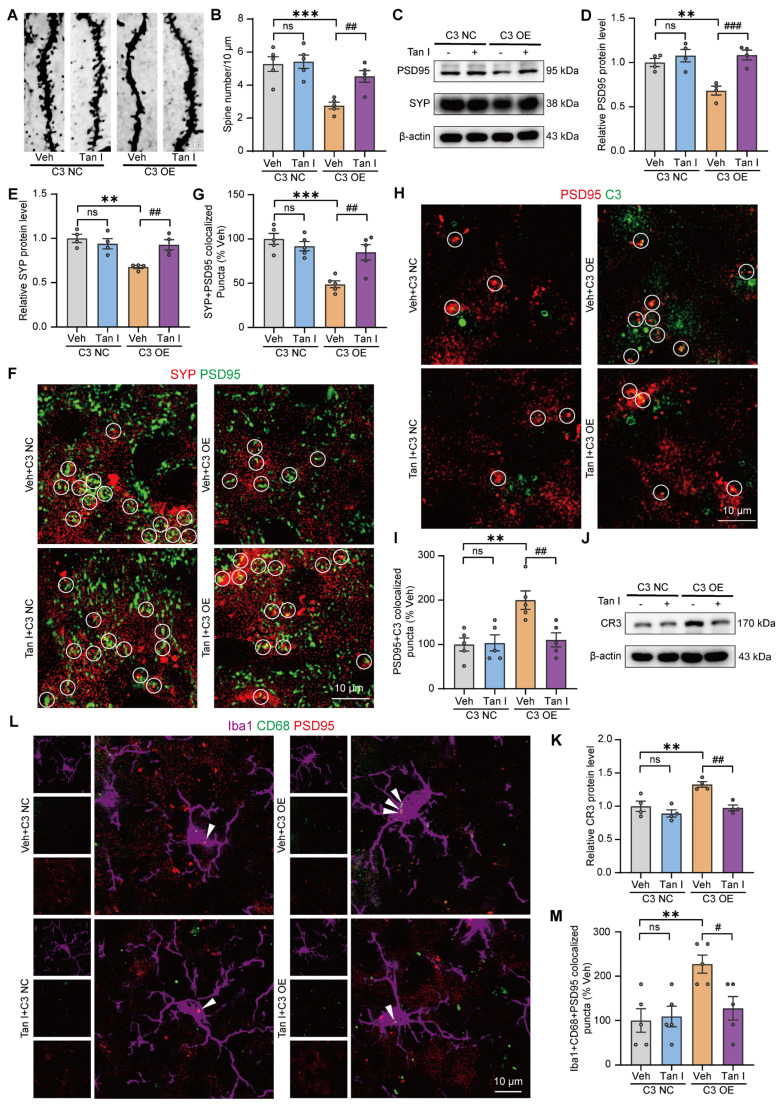
** Tan I inhibits microglia-mediated synaptic engulfment of C3 OE mice to exert antidepressant effects. (A-B)** The representative images **(A)** and dendritic spine density quantification **(B)** of Golgi-Cox staining in the mPFC (n = 5). Scale bar = 5 μm. **(C)** The levels of PSD95 and SYP in the mPFC were analyzed. **(D-E)** Relative quantification of PSD95 and SYP protein levels of C3 OE mice after Tan I treatment (n = 4). **(F)** Representative images of PSD95 (green) and SYP (red) in different groups of mice. Scale bar = 10 μm. Colocalized puncta were indicated by circles. **(G)** Quantification of the colocalization of SYP and PSD95 in different groups of mice (n = 5). **(H)** Representative images of PSD95 (green) and C3 (red) in different groups of mice. Scale bar = 10 μm. Colocalized puncta were indicated by circles. **(I)** Quantification of the colocalization of PSD95 and C3 of C3 OE mice after Tan I treatment (n = 5). **(J)** The levels of CR3 in the mPFC were analyzed. **(K)** Relative quantification of CR3 protein levels in different groups of mice (n = 4). **(L)** Representative images of Iba1^+^ microglia (purple) containing PSD95^+^ puncta (red) and CD68^+^ (green). White arrowheads showed the colocalization between PSD95 and CD68 in microglia. Scale bar = 10 μm. **(M)** Quantification of the colocalization of PSD95^+^ and CD68^+^ in Iba1^+^ microglia of C3 OE mice after Tan I treatment (n = 5). ^∗∗^*P* < 0.01, ^∗∗∗^*P* < 0.001 versus vehicle C3 NC group; ^#^*P* < 0.05, ^##^*P* < 0.01, ^###^*P* < 0.001 versus vehicle C3 OE group; ns, not significant. One-way ANOVA with Tukey's post hoc test. All data are expressed as mean ± SEM.

Additionally, the Golgi Staining analysis revealed that C3 OE mice exhibited a significant decrease in dendritic spine density in the mPFC relative to the C3 NC group, whereas Tan I treatment significantly increased the dendritic spine density compared to the C3 OE group (*F*_(3, 16)_ = 11.44, *P* < 0.001, Figure 7A-B). The results found that C3 OE mice exhibited a significant decrease in PSD95 and SYP levels in the mPFC relative to the C3 NC group, whereas Tan I treatment significantly increased PSD95 (*F*_(3, 12)_ = 11.54, *P* < 0.001) and SYP (*F*_(3, 12)_ = 8.898, *P* = 0.0022) levels compared to the C3 OE group (Figure 7C-E). Further, through immunofluorescence experiments, we observed that PSD95 and SYP were colocalized, and noted a significant loss of synapses in the mPFC of C3 OE mice. However, Tan I mitigated this synapse loss compared to the C3 OE group (*F*_(3, 16)_ = 13.03, *P* < 0.001, Figure 7F-G).

In addition, immunofluorescence images revealed a greater percentage of PSD95^+^ colocalized with C3 in the mPFC of C3 OE mice. The colocalization of PSD95^+^ with C3 was significantly reduced by Tan I, implying that Tan I reduces C3 deposition at synapses in C3 OE mice (*F*_(3, 16)_ = 7.420, *P* = 0.0025, Figure 7H-I). We investigated CR3 expression in the mPFC of C3 OE mice. The results indicated that CR3 levels were significantly elevated in C3 OE mice. However, Tan I reduced the CR3 increase of C3 OE mice (*F*_(3, 12)_ = 11.84, *P* < 0.001, Figure 7J-K). Further, we assessed the count of Iba1^+^ (red) and CR3^+^ (green) colocalized in the mPFC of C3 OE mice. The findings revealed a significant increase in the number of Iba1^+^ and CR3^+^ colocalization of C3 OE mice compared to the C3 NC group, which was reduced by Tan I (*F*_(3, 16)_ = 8.242, *P* = 0.0015, Figure S11A-B). Interestingly, the level of p-STAT3 expression was significantly higher in the mPFC of C3 OE mice, while Tan I significantly downregulated the level of p-STAT3 in the mPFC of C3 OE mice (*F*_(3, 12)_ = 15.59, *P* < 0.001; *F*_(3, 12)_ = 7.643, *P* = 0.004; Figure S12A-C). Furthermore, we performed immunofluorescence experiments, which showed that p-STAT3 expression was significantly upregulated in microglia in the mPFC of C3 OE mice, while Tan I inhibited this increase compared to the C3 OE group (*F*_(3, 16)_ = 15.20, *P* < 0.001, Figure S12D-E). These results suggest that Tan I inhibits C3 and affects the downstream STAT3 signaling pathway. Further, it was found that the proportion of PSD95 colocalized with CD68 was significantly higher in microglia from C3 OE mice. However, Tan I markedly decreased these colocalizations compared to the C3 OE group (*F*_(3, 16)_ = 5.848, *P* = 0.0068, Figure 7L-M). These findings imply that Tan I lessens synapse loss in C3 OE mice by inhibiting C3 deposition on synapses and microglia-induced synaptic engulfment.

### Tan I improves fMRI-based network changes in C3 OE mice

**Figure 8:**
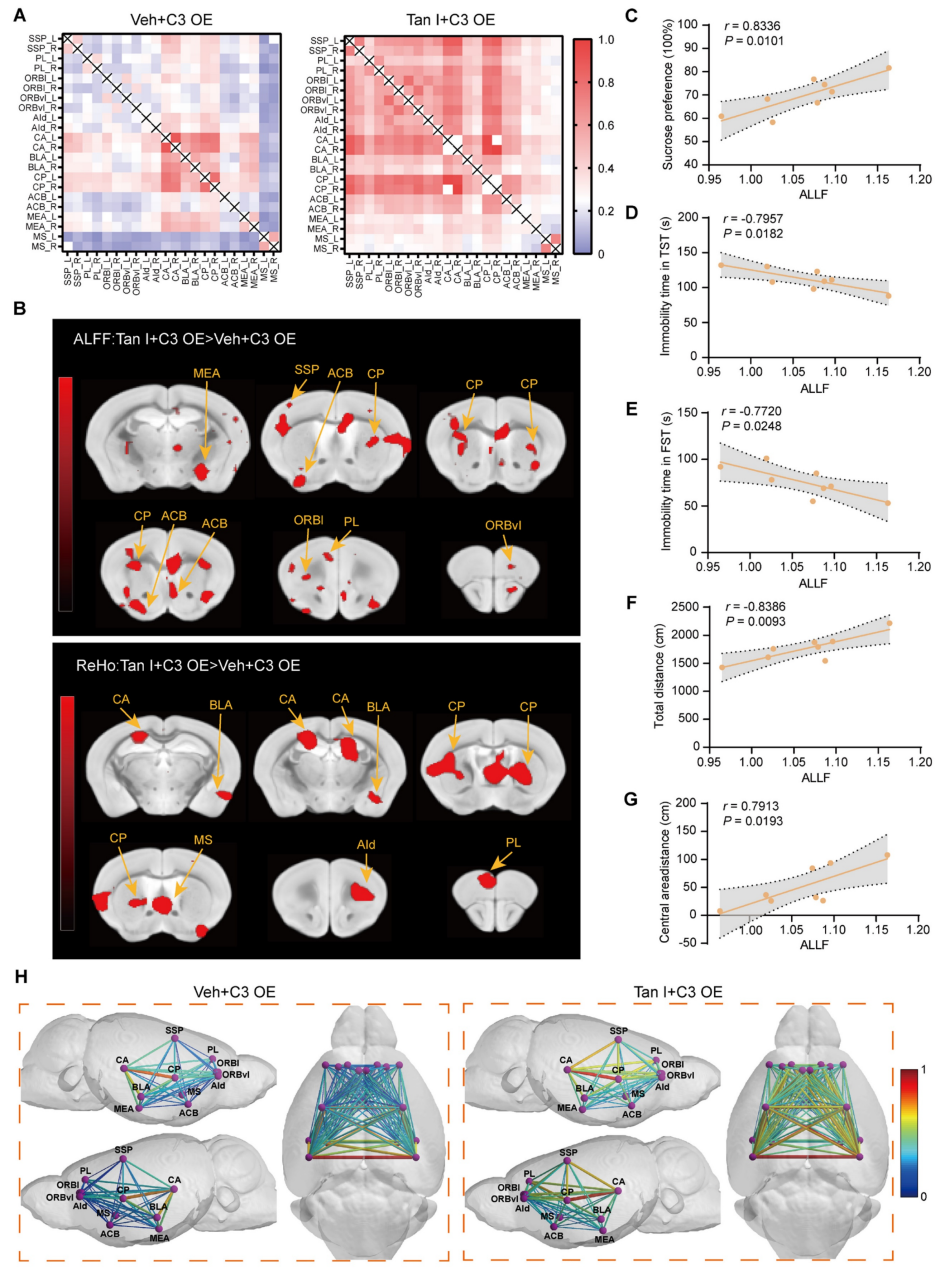
Tan I improves fMRI-based network changes in C3 OE mice. **(A)** The results of the heatmap showed that the Z-score matrices were lower in the Veh + C3 OE group of mice, whereas they increased after treatment with Tan I. **(B)** Comparative analysis of the ALFF and Reho values between the Tan I + C3 OE group and the Veh + C3 OE group. Different colors represent brain regions with significant differences based on Z-score values. **(C-G)** The correlation analysis between the behaviors with alterations in ALFF in PL. **(H)** The functional correlations between different brain regions are represented by lines, with redder colors and thicker lines indicating higher Z-score values. N = 4 mice.

We analyzed the differences in functional connectivity between the Tan I + C3 OE and Veh + C3 OE groups in mice. We conducted brain scans on C3 OE mice treated with Tan I and collected fMRI data. We conducted a region-specific analysis of fMRI data focusing on areas mainly impacted by depression 40, selecting ROIs such as the SSp, PL, ORBl, ORBvI, AId, CA, BLA, CP, ACB, MEA, and MS, for a total of 11 brain regions (consistent with those selected for CUS mice). MATLAB tools were used to process the fMRI data 41. The Z-score values were used to obtain the functional connectivity matrices for the Veh + C3 OE and Tan I+ C3 OE groups, with redder colors indicating stronger connectivity between regions. The Tan I + C3 OE group displayed a generally redder functional connectivity matrix compared to the Veh + C3 OE group, suggesting that Tan I treatment can improve fMRI-based network changes in C3OE mice (Figure 8A).

In addition, we analyzed ALFF and ReHO in the Veh + C3 OE group and the Tan I + C3 OE group to evaluate the overall effect of Tan I on brain activity in C3 OE mice. The voxel-level threshold for ALFF, without correction, was established at *P* < 0.01, with a cluster extent threshold of 20 voxels. Our results showed that ALFF levels were increased in the Tan I + C3 OE group compared to the Veh + C3 OE group, mainly in regions including the prelimbic region, the SSp, PL, ORBl, ORBvI, CP, ACB, and MEA (Figure 8B). For the ReHO analysis, the voxel-level threshold was also set to *P* <0.01, with a cluster range threshold of 10 voxels. We found that Reho levels were elevated in the Tan I + C3 OE group compared to the Veh + C3 OE group, mainly in brain regions including the PL, AId, CA, BLA, CP, and MS (Figure 8B).

We focused on alterations in the connectivity strength between PL and other regions associated with depression. The relationship between C3 OE mice behavior (SPT, OFT, TST, and FST) and ALFF values of PL was explored by correlation analysis. According to the study results, there was a notable correlation between the behavioral indices and the ALFF value of PL in C3 OE mice (Figure 8C-G). Notably, the ALFF value of PL had a negative correlation with the immobilization time in both the TST and FST in C3 OE mice (Figure 8D-E).

Further, we visualized the data using lines to show the connections between specific brain regions, with redder colors and thicker lines signifying stronger connectivity. In the Veh + C3 OE group, the lines appeared bluer and thinner, but following Tan I treatment, they became redder and thicker (Figure 8H), suggesting improved connectivity. These findings imply that Tan I treatment can enhance the functional connectivity among the brain regions in C3 OE mice.

All of the above findings indicate that Tan I treatment improves functional connectivity between brain regions in C3 OE mice. These findings highlight the crucial role of complement C3 in fMRI-based network changes. Therefore, Tan I improves fMRI-based network changes and depression-like behavior in C3 OE mice.

### Tan I does not improve depressive-like behaviors in C3mut OE mice

In addition, we mutated the four amino acid sites LYS 1084, LEU 1088, GLN 1277, and GLN 1281, where Tan I and C3 are most likely to bind, based on the previous MD simulations, to construct a C3 mutant (C3mut) (K1084G, L1088F, Q1277G, Q1281A) lentivirus. Similarly, as shown in Figure S13A, we injected lentivirus-mediated C3mut overexpression bilaterally into the PFC of WT mice. The results of PCR (*F*_(3, 12)_ = 8.511, *P* = 0.0027, Figure S13B) and Western blot (*F* (3, 12) = 34.23, *P* < 0.001, Figure S13C-D) showed that the levels of C3 were significantly elevated in the mPFC of the C3mut OE mice compared with the C3 NC mice. In contrast, Tan I treatment failed to significantly reduce the C3 levels compared to the C3mut OE mice. Notably, C3mut OE mice exhibited depressive-like behaviors. However, we found that administering 90 mg/kg of Tan I failed to improve depression-like behaviors in the SPT, TST, FST, and OFT in C3mut OE mice (*F*_(3, 28)_ = 18.37, *P* < 0.001, Figure S13E; *F*_(3, 28)_ = 24.92, *P* < 0.001, Figure S13F; *F*_(3, 28)_ = 16.20, *P* < 0.001, Figure S13G; *F*_(3, 28)_ = 10.73, *P* < 0.001; *F*_(3, 28)_ = 11.37, *P* < 0.001, Figure S13H-J), suggesting that Tan I exerts its antidepressant effect by binding to and inhibiting C3, and the binding sites were LYS 1084, LEU 1088, GLN 1277, and GLN 1281.

**Figure 9:**
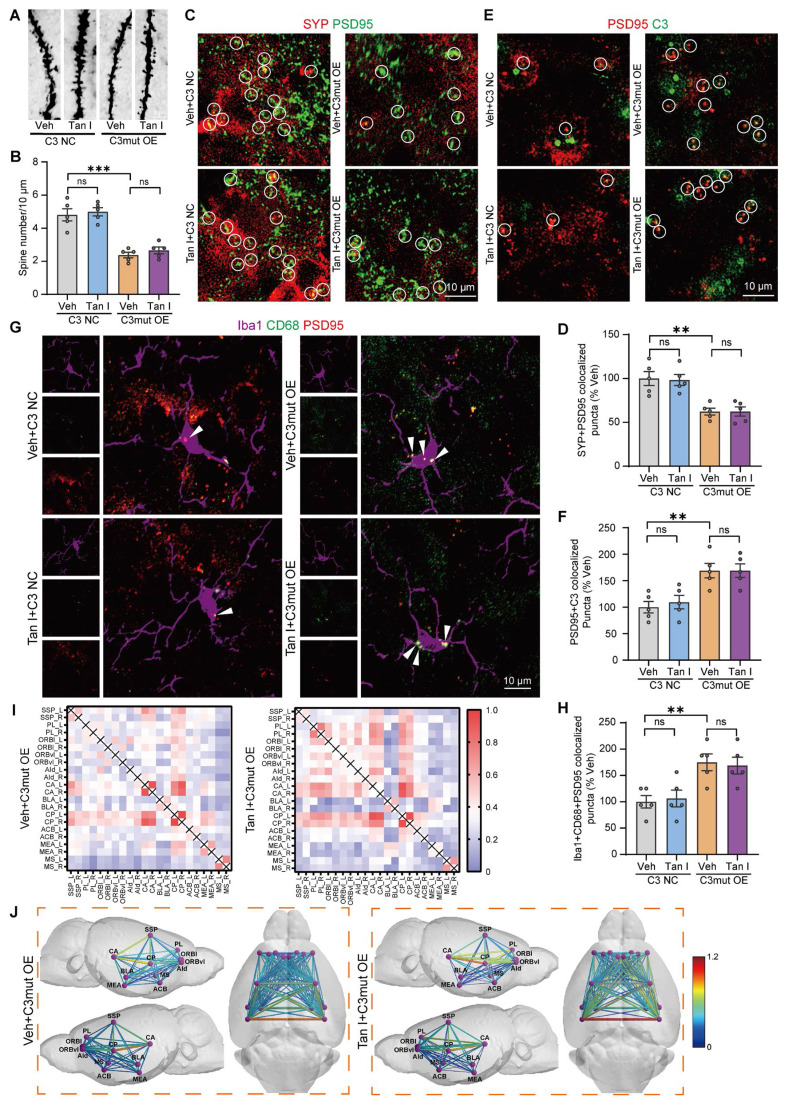
** Tan I does not inhibit microglia-mediated synaptic engulfment or improve fMRI-based network changes in C3mut OE mice. (A-B)** The representative images **(A)** and dendritic spine density quantification **(B)** of Golgi-Cox staining in the mPFC (n = 5). Scale bar = 5 μm. **(C)** Representative images of PSD95 (green) and SYP (red) in different groups of mice. Scale bar = 10 μm. Colocalized puncta were indicated by circles. **(D)** Quantification of the colocalization of SYP and PSD95 in different groups of mice (n = 5). **(E)** Representative images of PSD95 (green) and C3 (red) in different groups of mice. Scale bar = 10 μm. Colocalized puncta were indicated by circles. **(F)** Quantification of the colocalization of PSD95 and C3 of C3mut OE mice after Tan I treatment (n = 5). **(G)** Representative images of Iba1^+^ microglia (purple) containing PSD95^+^ puncta (red) and CD68 (green). White arrowheads showed the colocalization between PSD95 and CD68 in microglia. Scale bar = 10 μm. **(H)** Quantification of the colocalization of PSD95^+^ and CD68^+^ in Iba1^+^ microglia of C3mut OE mice after Tan I treatment (n = 5). **(I)** The heat map results indicated no significant difference in the Z-score matrix between the Tan I + C3mut OE mice and the Veh + C3mut OE mice. **(J)** The functional correlations between different brain regions are represented by lines, with redder colors and thicker lines indicating higher Z-score values. N = 4 mice. ^∗∗^*P* < 0.01, ^∗∗∗^*P* < 0.001 versus vehicle C3 NC group; ns, not significant. One-way ANOVA with Tukey's post hoc test. All data are expressed as mean ± SEM.

Additionally, the Golgi Staining analysis revealed that C3mut OE mice exhibited a significant decrease in dendritic spine density in the mPFC relative to the C3 NC group, whereas Tan I treatment did not significantly increase the dendritic spine density compared to the C3mut OE group (*F*_(3, 16)_ = 28.48, *P* < 0.001, Figure 9A-B). We conducted a Western blot analysis to determine if Tan I treatment could affect PSD95 (*F*_(3, 12)_ = 13.41, *P* < 0.001) and SYP (*F*_(3, 12)_ = 5.745, *P* = 0.0113) levels in the mPFC of C3mut OE mice. We found that Tan I could not significantly increase PSD95 and SYP levels compared to the C3mut OE group (Figure S14A-C).

Further, through immunofluorescence experiments, we observed that PSD95 and SYP were colocalized, and noted a significant loss of synapses in the mPFC of C3mut OE mice. However, Tan I did not attenuate this synaptic loss compared to the C3mut OE group (*F*_(3, 16)_ = 12.52, *P* < 0.001, Figure 9C-D). Subsequently, we examined the colocalization of C3 and PSD95 in the mPFC of C3mut OE mice. Our immunofluorescence images revealed a greater percentage of PSD95^+^ colocalized with C3 in the mPFC of C3mut OE mice. However, the colocalization of PSD95^+^ with C3 was not significantly reduced by Tan I, implying that Tan I could not reduce C3 deposition at synapses in C3mut OE mice (*F*_(3, 16)_ = 8.774, *P* = 0.0011, Figure 9E-F). We investigated CR3 expression in the mPFC of C3mut OE mice. The results indicated that CR3 levels were significantly elevated in C3mut OE mice. However, Tan I failed to reduce the CR3 increase in the mPFC of C3mut OE mice (*F*_(3, 12)_ = 12.41, *P* < 0.001, Figure S14D-E). Further, we assessed the count of Iba1^+^ (red) and CR3^+^ (green) colocalized in the mPFC of C3mut OE mice. The results indicated a significant increase in the number of Iba1^+^ and CR3^+^ colocalization in the mPFC of C3mut OE mice compared to the C3 NC group, while Tan I failed to reduce these increases (*F*_(3, 16)_ = 7.399, *P* = 0.0025, Figure S14F-G). The Western blot results showed that the level of p-STAT3 expression was significantly higher in the mPFC of C3mut OE mice, while Tan I failed to significantly downregulate p-STAT3 levels in the mPFC of C3mut OE mice (*F*_(3, 12)_ = 9.767, *P* = 0.0015; *F*_(3, 12)_ = 27.95, *P* < 0.001, Figure S15A-C). Further, we performed immunofluorescence experiments, which showed that p-STAT3 expression was significantly upregulated in microglia in the mPFC of C3mut OE mice. However, Tan I failed to inhibit this increase compared to the C3mut OE group (*F*_(3, 16)_ = 17.77, *P* < 0.001, Figure S15D-E). Additionally, we examined the colocalization of PSD95 and CD68 in microglia in the mPFC of C3mut OE mice. Our findings indicated that the proportion of PSD95 colocalized with CD68 was significantly higher in microglia from C3mut OE mice. Nonetheless, Tan I failed to markedly decrease these colocalizations compared to the C3mut OE group (*F*_(3, 16)_ = 7.072, *P* = 0.0031, Figure 9G-H).

We analyzed the differences in functional connectivity between the Tan I + C3mut OE and Veh + C3mut OE groups in mice. We conducted brain scans on C3mut OE mice treated with Tan I and collected fMRI data. We conducted a region-specific analysis of fMRI data focusing on areas mainly impacted by depression 40, selecting ROIs such as the SSp, PL, ORBl, ORBvI, AId, CA, BLA, CP, ACB, MEA, and MS, for a total of 11 brain regions (consistent with those selected for CUS mice). MATLAB tools were used to process the fMRI data 41. The Z-score values were used to obtain the functional connectivity matrices for the Veh + C3mut OE and Tan I + C3mut OE groups, with redder colors indicating stronger connectivity between regions. Notably, there was no significant change in the functional connectivity matrix in the Tan I + C3mut OE group compared to the Veh + C3mut OE group, suggesting that Tan I treatment was unable to improve fMRI-based network changes in C3mut OE mice (Figure 9I). Further, we visualized the data using lines to show the connections between specific brain regions, with redder colors and thicker lines signifying stronger connectivity. Notably, there was no significant change in the color and thickness of the lines in the Tan I + C3mut OE group compared to the Veh + C3mut OE group, suggesting that Tan I treatment was unable to improve the functional connectivity among the brain regions in C3mut OE mice (Figure 9J).

All of the above findings demonstrate that Tan I was unable to improve depression-like behavior, inhibit microglia-mediated synaptic engulfment, or improve fMRI-based network changes in C3mut OE mice, suggesting that Tan I exerts its antidepressant effect by binding to and thus inhibiting C3 and that the binding sites are LYS 1084, LEU 1088, GLN 1277, and GLN 1281.

## Discussion

This study demonstrates that Tan I improved depression-like behaviors in CUS mice. Additionally, it was found that Tan I inhibited the reduction of synaptic loss and neuronal activity and reduced microglial activation and phagocytic activity in the mPFC of CUS mice. The DARTS and MST revealed that the specific target of Tan I is complement C3. Furthermore, Tan I decreased the CUS-induced synaptic loss by inhibiting the deposition of C3 deposition onto synapses and subsequent microglia-mediated synaptic engulfment. Importantly, Tan I also improved fMRI-based network changes in CUS mice. Overexpression of C3 in the mPFC of normal mice leads to depressive-like behavior, accompanied by synaptic loss and reduced fMRI-based network changes. In contrast, administration of Tan I inhibits microglia-mediated synaptic phagocytosis and improves fMRI-based network changes, which in turn ameliorate the depressive-like behaviors in C3-overexpressing mice. Collectively, the study demonstrated that Tan I acts as a potent natural C3 modulator that binds directly to C3, blocks the C3-CR3 axis and downstream STAT3 signaling pathway, inhibits microglia-mediated synaptic engulfment, and improves fMRI-based network changes, which in turn exert antidepressant effects.

It has been shown that stress induces oxidative stress and neuroinflammation in the brain 42, and stress is also a significant risk factor for psychiatric disorders. Since Tan I has excellent anti-oxidant and anti-inflammatory effects 21, this raises the question of whether Tan I has antidepressant effects. Indeed, we found that Tan I significantly improved depression-like behaviors in CUS mice at effective doses of 30 mg/kg and 90 mg/kg, suggesting that Tan I has significant antidepressant effects. In addition, the effect of Tan I at 30 mg/kg was comparable to that of Flu at 10 mg/kg, while the effect of Tan I at 90 mg/kg was more significant. Additionally, we observed that CUS mice altered synapse number and dendritic spine complexity in the mPFC, which was in agreement with prior research 43. Tan I enhanced the number of synapses and density of dendritic spines in CUS mice. Additionally, our results indicated that CUS mice significantly reduced the activity of excitatory neurons in the mPFC, while Tan I significantly increased the activity of these neurons.

Numerous studies have indicated that stress activates microglia in the hippocampus and prefrontal cortex of mice 44. In line with this, we found that microglia were activated in the mPFC of CUS mice. It has been shown that when microglia are activated, they release oxidative products and pro-inflammatory mediators such as TNF-α and IL-1β, which facilitate neuronal damage and contribute to depression 28. In line with earlier research, we found that IL-1β and TNF-α levels were notably elevated in CUS mice, but Tan I significantly reduced these increases. In addition, the results of immunofluorescence showed a significant increase in the number of Iba1^+^ and CD68^+^ colocalization in the mPFC of CUS mice, which was reduced by Tan I, suggesting that Tan I reduces the phagocytic activity of microglia in CUS mice.

Further, we performed a DARTS assay to screen and identify the specific targets of Tan I, and the results indicated that C3 might be a specific target of Tan I. We conducted a Western blot to further confirm this discovery, showing that Tan I protected C3 from proteolytic degradation. In addition, CETSA results showed that Tan I significantly enhances the thermal stability of C3 in a certain temperature range, confirming the specific interaction between Tan I and C3. Furthermore, the results of MST experiments indicated a direct interaction between Tan I and C3 with a KD value of 3.75 ± 3.31 µM. All these results suggest a direct interaction between Tan I and C3. To further uncover the interaction and binding site of Tan I with C3, an MD simulation was employed, and the amino acids predicted to be the top contributors to the binding of Tan I and C3, specifically LYS 1084, LEU 1088, GLN 1277, and GLN 1281. These amino acids are thus critical for Tan I and C3 binding. All the above results show that Tan I and C3 can directly bind to their specific target, and the binding site may be LYS 1084, LEU 1088, GLN 1277, and GLN 1281 amino acids.

In both healthy and diseased brains, recently, microglia have been acknowledged as a key factor in regulating synaptic connectivity through microglia-mediated synapse elimination 12. In the brain, the complement system is crucial for regulating microglial synaptic pruning. In the developing mouse brain, complement C3 and C1q target synapse subsets and are required for microglia to eliminate these synapses 13,14. The growth of chemotactic microglia is directed by complement C3, which also binds to the complement receptor (CR3) on microglia, prompting microglia to engulf labeled synapses. In this study, we discovered that, similar to C1q being found at synapses in a mouse model of Alzheimer's disease (AD) 34, C3 is also located at synapses in the mPFC of CUS mice. Significantly, we found that the proportion of PSD95 colocalized with CD68 was significantly higher in microglia from CUS mice. Nonetheless, Tan I markedly decreased these colocalizations. These results indicate that Tan I plays a protective role in synaptic phagocytosis mediated by microglia in CUS mice.

Studies have shown that synaptic loss is associated with fMRI-based network change abnormalities in depression 17. In adults with MDD, fMRI studies have demonstrated disrupted functional connectivity in brain networks underlying its symptomatic expression 18,19. Therefore, it is crucial to investigate the effects of complement-mediated synaptic loss on fMRI-based network changes in depression. Consequently, we analyzed the differences in functional connectivity between the Tan I + CUS and Veh + CUS groups in mice. Our results imply that Tan I treatment can enhance functional connectivity among the brain regions in CUS mice. The relationship between CUS mice behavior (SPT, OFT, TST, and FST) and ALFF values of PL was explored by correlation analysis. According to the study results, there was a notable correlation between the behavioral indices and the ALFF value of PL in CUS mice. The findings offer further neuroimaging evidence that Tan I improves depressive-like behaviors. In addition, we analyzed ALFF and ReHO in the Veh + CUS group and the Tan I + CUS group to evaluate the overall effect of Tan I on brain activity in CUS mice. All of the above findings indicate that Tan I treatment improves functional connectivity between brain regions in CUS mice.

Complement protein C3 plays a crucial role in the complement system and is vital for immune regulation, as well as influencing depression development. Research indicates an elevation in C3 levels in the postmortem brains of those with depression and mice models related to stress 45. Here, we found that C3 levels were elevated in the mPFC of CUS mice. However, Tan I significantly reduced the increase of C3 in the mPFC of CUS mice, and the inhibitory effect of Tan I on C3 was dose-dependent. In addition, the activation of the C3-CR3 axis has been shown to play an essential role in microglia-mediated phagocytosis of synapses. Complement C3 induces microglia to engulf labeled synapses by binding to CR3 receptors on chemotactic microglia. Therefore, we investigated whether Tan I blocks the interaction between C3 and its receptor, CR3, through MST assays. The results of MST showed that the Kd value of C3 and CR3 was calculated to be 5.73 ± 10.8 nM, while the Kd value of the C3-Tan I complex and CR3 was calculated to be 74.05 ± 24.31 nM. This indicates that Tan I and CR3 competitively bind to C3 and that Tan I can block the interaction between C3 and its receptor, CR3. The STAT3 signaling pathway is an important downstream pathway activated by C3. When STAT3 is activated in microglia, it promotes their transformation into reactive microglia, which then perform several functions, such as synaptic pruning. To explore the potential downstream pathways through which Tan I inhibits the effects of C3, we investigated the STAT3 signaling pathway. Interestingly, the level of p-STAT3 expression was significantly higher in the mPFC of CUS mice, while Tan I significantly downregulated the level of p-STAT3 in the mPFC of CUS mice. Similarly, immunofluorescence experiments showed that p-STAT3 expression was significantly upregulated in microglia in the mPFC of CUS mice, while Tan I inhibited this increase compared to the CUS group. These results suggest that Tan I inhibits C3 and affects the downstream STAT3 signaling pathway.

Significantly, we found that LV-C3 injection in the mPFC caused depressive-like behaviors, which were improved with Tan I treatment, indicating that Tan I could act as an antidepressant by inhibiting C3 expression in the mPFC. Here, we observed that C3 OE mice altered synapse number and dendritic spine complexity in the mPFC. Tan I enhanced the number of synapses and density of dendritic spines in C3 OE mice. Interestingly, the level of p-STAT3 expression was significantly higher in the mPFC of C3 OE mice, while Tan I significantly downregulated the level of p-STAT3 in the mPFC of C3 OE mice. Similarly, immunofluorescence experiments showed that p-STAT3 expression was significantly upregulated in microglia in the mPFC of C3 OE mice, while Tan I inhibited this increase compared to the C3 OE group. Significantly, we found that the proportion of PSD95 colocalized with CD68 was significantly higher in microglia from C3 OE mice. Nonetheless, Tan I markedly decreased these colocalizations. These findings imply that Tan I lessens synapse loss in C3 OE mice by inhibiting C3 deposition on synapses and microglia-induced synaptic engulfment. Further, we analyzed the differences in functional connectivity between the Tan I + C3 OE and Veh + C3 OE groups in mice. Our findings imply that Tan I treatment can enhance the functional connectivity among the brain regions in C3 OE mice. These findings highlight the crucial role of complement C3 in fMRI-based network changes. Therefore, Tan I improves fMRI-based network changes and depression-like behavior in C3 OE mice.

Notably, C3mut OE mice exhibited depressive-like behaviors. However, we found that administering 90 mg/kg of Tan I failed to improve depression-like behaviors in the SPT, TST, FST, and OFT in C3mut OE mice. Here, we observed that C3mut OE mice altered synapse number and dendritic spine complexity in the mPFC, whereas Tan I treatment did not significantly increase the number of synapses and density of dendritic spines in C3mut OE mice. Similarly, the level of p-STAT3 expression was significantly increased in the mPFC of C3mut OE mice, while Tan I failed to significantly downregulate the level of p-STAT3 in the mPFC of C3mut OE mice. Additionally, we found that the proportion of PSD95 colocalized with CD68 was significantly higher in microglia from C3mut OE mice. Nonetheless, Tan I failed to markedly decrease these colocalizations. Further, we analyzed the differences in functional connectivity between the Tan I + C3mut OE and Veh + C3mut OE groups in mice. Our findings imply that Tan I treatment was unable to improve fMRI-based network changes in C3mut OE mice. All of the above findings demonstrate that Tan I was unable to improve depression-like behavior, inhibit microglia-mediated synaptic engulfment, or improve fMRI-based network changes in C3mut OE mice, suggesting that Tan I exerts its antidepressant effect by binding to and thus inhibiting C3 and that the binding sites are LYS 1084, LEU 1088, GLN 1277, and GLN 1281.

Studies have shown that significant increases in C3 mRNA levels were found in the prefrontal cortex of depressed suicide patients^45^. In addition, postmortem studies have shown that the loss of postsynaptic proteins, such as PSD95, has been observed in the PFC with major depressive disorder^46^. The above studies suggest that C3 may play an important role in synaptic loss in depression. Our study also supports the conclusion that C3-mediated synaptic loss produces depressive-like behaviors. While this study provides compelling evidence that Tan I binds directly to C3, inhibiting microglia-mediated synaptic engulfment and improving fMRI-based network changes, there are limitations. Currently, only male mice were used in this study, and to determine whether there are gender-specific differences in the antidepressant effects and mechanisms of Tan I, it will be necessary to include female mice in future experiments. In addition, whether Tan I also impacts other complement pathways or non-microglial cells remains to be further studied. Although we identified the Tan I binding sites as LYS 1084, LEU 1088, GLN 1277, and GLN 1281, we will further investigate mutating one or more of these sites in the future. In addition, in terms of mice behavior, the effect of Tan I at 30 mg/kg was comparable to that of Flu at 10 mg/kg in improving depression-like behaviors in CUS mice, while the effect of Tan I at 90 mg/kg was more significant. In terms of rescuing synaptic loss, compared with the CUS group, Tan I at doses of 30 mg/kg and 90 mg/kg both improved synaptic loss in CUS mice, whereas the dose of 10 mg/kg failed to improve synaptic loss. In addition, Tan I at 30 mg/kg was comparable to Flu at 10 mg/kg in improving synaptic loss in CUS mice, while Tan I showed more significant effects at 90 mg/kg. However, this study only compared the efficacy of Tan I and Flu in CUS mice subjected to continuous stress for 8 weeks. Further research is necessary to investigate the speed of their onset of action. Structural modifications of Tan I could be made in the future to reduce its dosage and further enhance its antidepressant effects in clinical settings.

**Figure 10:**
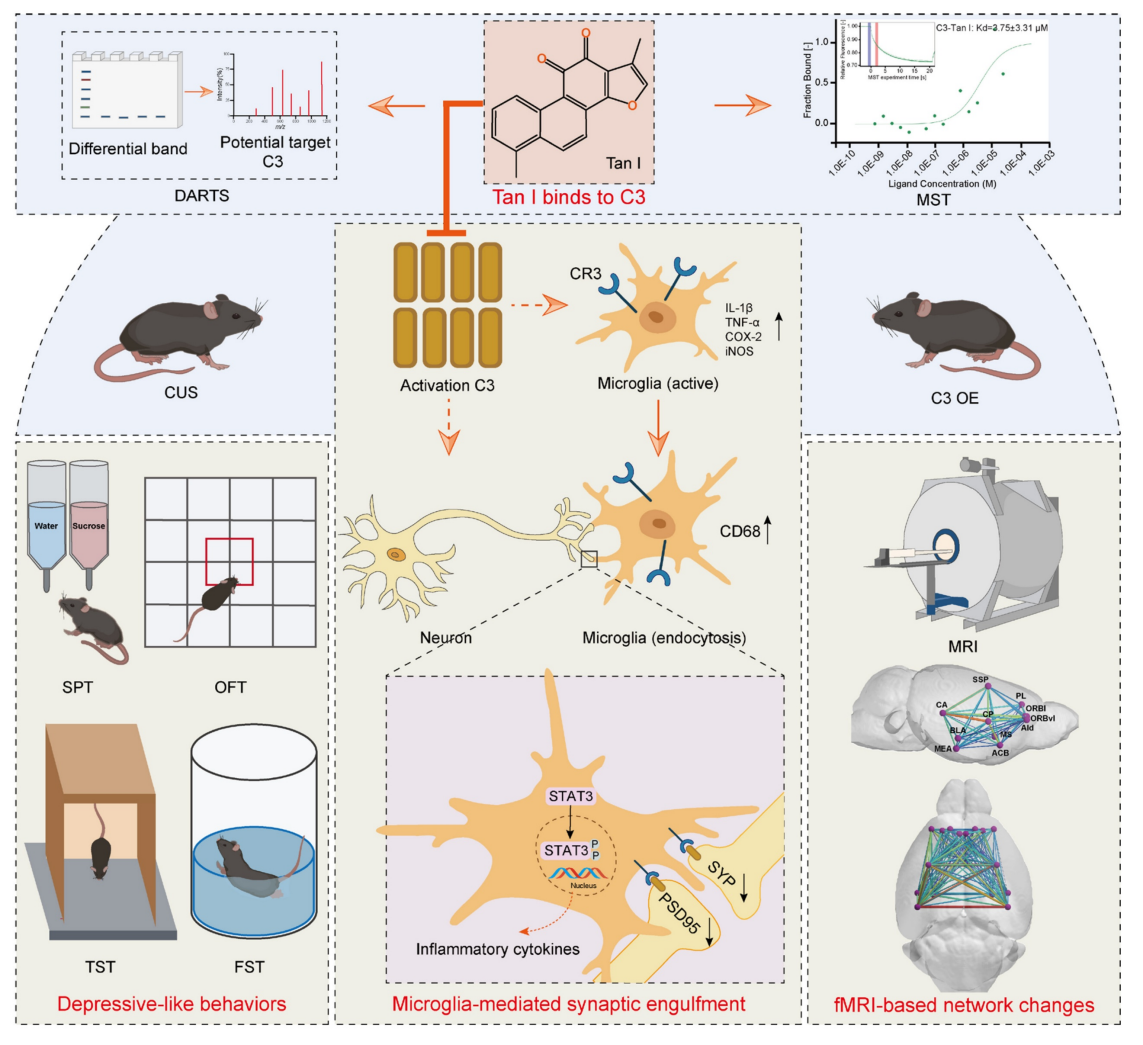
** Targeting complement C3 with Tan I decreases microglia-mediated synaptic engulfment to exert antidepressant effects**. Tan I acts as a potent natural C3 modulator that binds directly to C3, blocks the C3-CR3 axis and downstream STAT3 signaling pathway, inhibits microglia-mediated synaptic engulfment, and improves fMRI-based network changes, which in turn exert antidepressant effects.

In conclusion, we demonstrated that Tan I acts as a potent natural C3 modulator that binds directly to C3, blocks the C3-CR3 axis and downstream STAT3 signaling pathway, inhibits microglia-mediated synaptic engulfment, and improves fMRI-based network changes, which in turn exert antidepressant effects (Figure 10). Our research indicates that Tan I could be an effective natural compound for treating depression. These discoveries form a theoretical underpinning for Tan I antidepressant drug research and its clinical implementation.

## Supplementary Material

Supplementary figures and tables.

## Abbreviations

ALFF: abnormal low-frequency fluctuation
BBB: blood-brain barrier
BLA: basolateral amygdala
CA: cornu ammonis
CCK-8: cell counting Kit-8
CETSA: cellular thermal shift assay
CORT: corticosterone
CUS: chronic unpredictable stress
DARTS: drug affinity responsive target stability
DEGs: differentially expressed genes
DMEM: Dulbecco's modified eagle medium
ELISA: enzyme-linked immunosorbent assay
Flu: fluoxetine
fMRI: functional magnetic resonance imaging
FST: forced swimming test
GSEAs: gene set enrichment analyses
HE: Hematoxylin and eosin
IL-1β: interleukin-1β
KEGG: Kyoto Encyclopedia of Genes and Genomes
LC-MS/MS: liquid chromatography-tandem mass spectrometry
LV: lentiviral
MD: molecular dynamics
MDA: malondialdehyde
mPFC: medial prefrontal cortex
MST: microscale thermophoresis
OFT: open field test
PL: prelimbic
PSD95: postsynaptic density 95
ReHO: regional homogeneity
ROIs: regions of interest
SPT: sucrose preference test
STAT3: signal transducer and activator of transcription 3
SYP: synaptophysin
Tan I: Tanshinone I
TNF-α: tumor necrosis factor-α
TST: tail suspension test
