# Supplementary material for: Targeting complement C3 with Tanshinone I decreases microglia-mediated synaptic engulfment to exert antidepressant effects
Source: Theranostics. 2025 Jul 24;15(16):8150–75. doi: 10.7150/thno.115587 (PMC12374583; doi:10.7150/thno.115587)
Supplement: Supplementary file 1 — Supplementary figures and tables. [file thnov15p8150s1.pdf]

Supplementary material

Targeting complement C3 with Tanshinone I decreases microglia-mediated synaptic engulfment to exert antidepressant effects

Huaqing Lai [1], Pinglong Fan [1], Pengxiang Zhang [1], Meng Zhang [2], Xinmu Li [2], Boyu Kuang [3], Run Zhou [4], Wenfei Wang [3], Hong Jiang [2], Zhenzhen Wang [2*], Naihong Chen [1,2,3*]

*Corresponding author. Zhenzhen Wang (E-mail: wangzz@imm.ac.cn) and Naihong Chen (E-mail: chennh@imm.ac.cn).

This file includes:

Supplementary figures: Figure S1 to S15.

Supplementary tables: Table S1 to S2.

Supplementary figures

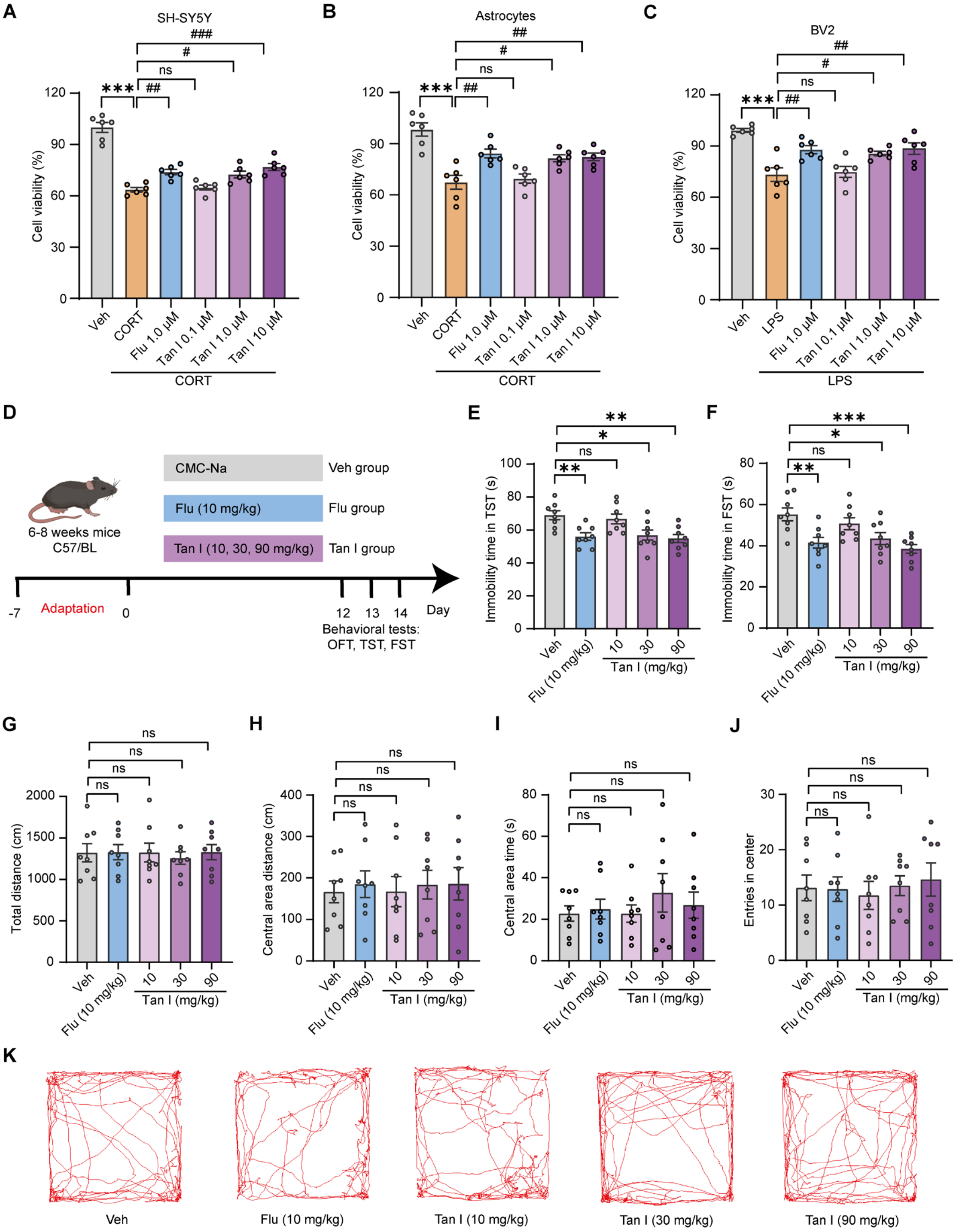

Figure S1. Tan I exhibits significant antidepressant potential. (A-C) The effect of different concentrations of Tan I on the viability of SH-SY5Y cells **(A)**, primary astrocytes **(B)**, and BV2 cells **(C)** (n = 6). The survival rate of the cells was significantly enhanced by Tan I treatment, suggesting that Tan I has excellent antidepressant potential. **(D)** Schematic timeline of experimental procedures. **(E-F)** Flu (10 mg/kg) or Tan I (10, 30, or 90 mg/kg) reduced the immobility time in the

TST **(E)** and FST **(F)** in a dose-dependent manner (n = 8). Tan I (30 and 90 mg/kg) and Flu (10 mg/kg) significantly decreased the immobility time of mice in TST and FST, implying that Tan I has antidepressant effects at certain doses, with effective doses being 30 mg/kg and 90 mg/kg. **(G-J)** Tan I (10, 30, or 90 mg/kg) or Flu (10 mg/kg) did not affect the total distance **(G)**, the distance in the center **(H)**, the time spent in the center **(I)**, or the number of entries into the center **(J)** in the OFT (n = 8). This suggests that the effects of Tan I on TST and FST are not related to spontaneous activity in mice. **(K)** Representative traces of mice movement in the OFT. $^{**}P < 0.01$, $^{***}P < 0.001$ versus vehicle group; $^{\#}P < 0.05$, $^{\#\#}P < 0.01$, $^{\#\#\#}P < 0.001$ versus model group; ns, not significant. One-way ANOVA with the Tukey's post hoc test. All data are expressed as mean ± SEM.

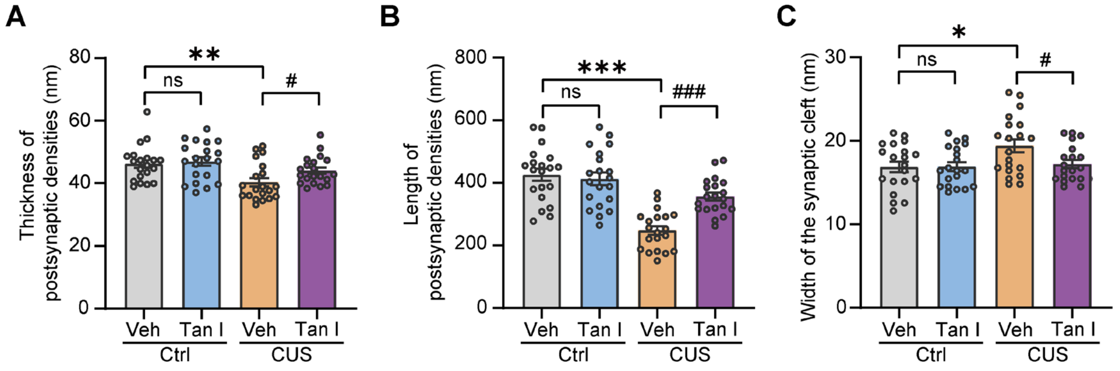

Figure S2. Tan I improves the neuronal structure in the mPFC of CUS mice. (A-B) The thickness (**A**) and length **(B)** of postsynaptic density were calculated (n = 20 synapses from 5 mice per group). **(C)** The synaptic cleft width was calculated (n = 20 synapses from 5 mice per group). $^{*}P < 0.05$, $^{**}P < 0.01$, $^{***}P < 0.001$ versus vehicle control group; $^{\#}P < 0.05$, $^{\#\#\#}P < 0.001$ versus vehicle CUS group; ns, no significant.

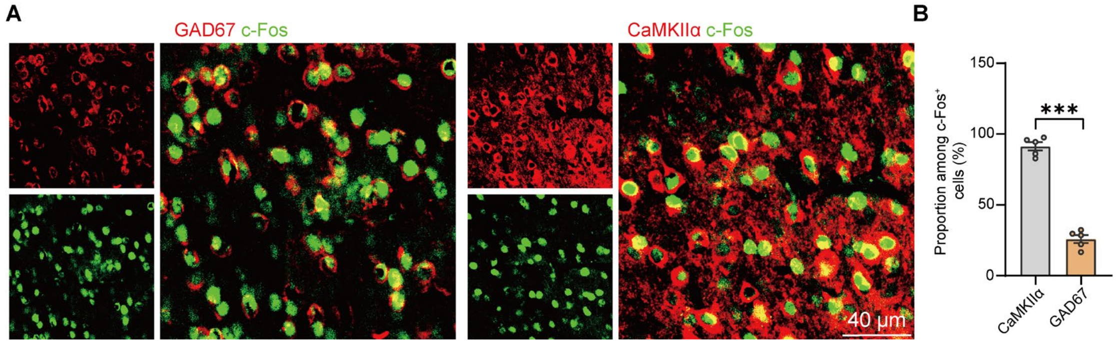

Figure S3. Excitatory neurons were predominantly activated in the mPFC of mice after FST. (A) Representative images of GAD67 (red) or CaMKIIα (red) positive cells colocalized with c-Fos (green) positive cells of the vehicle control group mice. Scale bar = 40 μm. **(B)** Quantification of GAD67 or CaMKIIα and c-Fos double-positive cells of vehicle control group mice (n = 5). ***$P < 0.001$. Two-tailed Student's t test. All data are expressed as mean ± SEM.

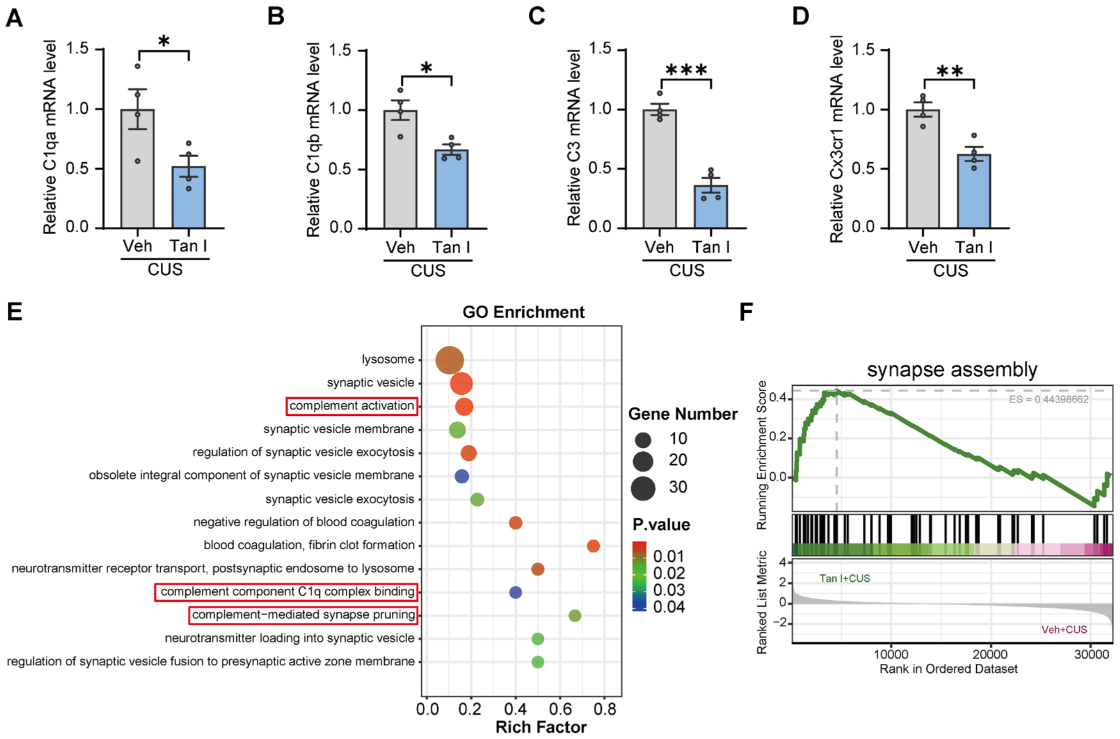

Figure S4. The Tan I treatment altered the gene expression profile in the mPFC tissues of CUS mice. (A-D) The expression levels of the C1qa **(A)**, C1qb **(B)**, C3 **(C)**, and Cx3cr1 **(D)** genes were verified by qPCR. **(E)** GO enrichment analysis for DEGs in the mPFC tissues. (**F**) GSEA analyses indicated that the processes of synapse assembly were changed. **P* < 0.05, ***P* < 0.01, ****P* < 0.001 versus vehicle CUS group. Two-tailed Student's t test. All data are expressed as mean ± SEM.

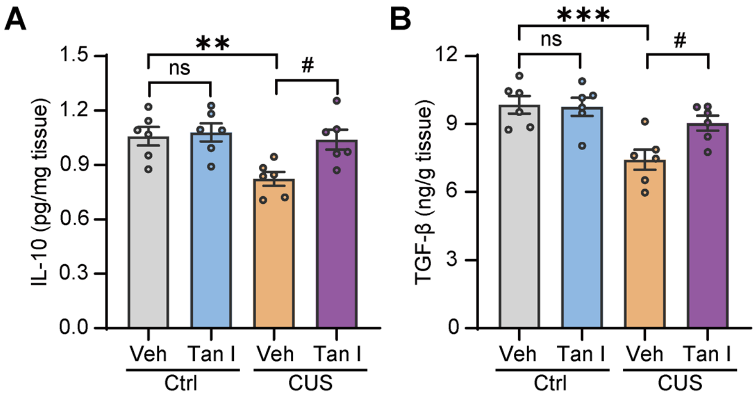

Figure S5. Tan I inhibits neuroinflammation in CUS mice. (A-B) The levels of IL-10 (**A**) and TGF-β (**B**) were determined in the mPFC tissues in different groups of mice (n = 4). $^{**}P < 0.01$, $^{***}P < 0.001$ versus vehicle control group; $^{\#}P < 0.05$ versus vehicle CUS group; ns, not significant. One-way ANOVA with the Tukey's post hoc test. All data are expressed as mean ± SEM.

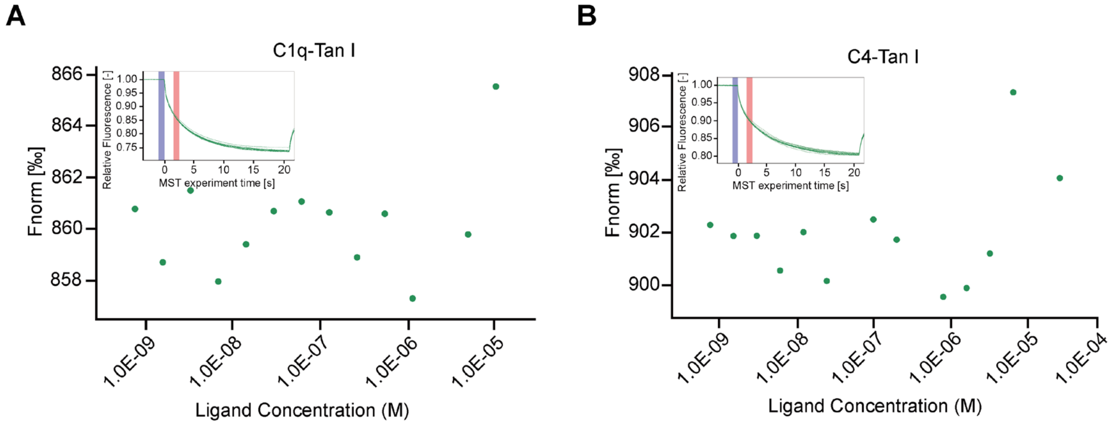

Figure S6. MST was used to analyze the binding of C1q and C4 to Tan I. (A) MST result showing no interaction between C1q and Tan I. **(B)** MST result showing no interaction between C4 and Tan I.

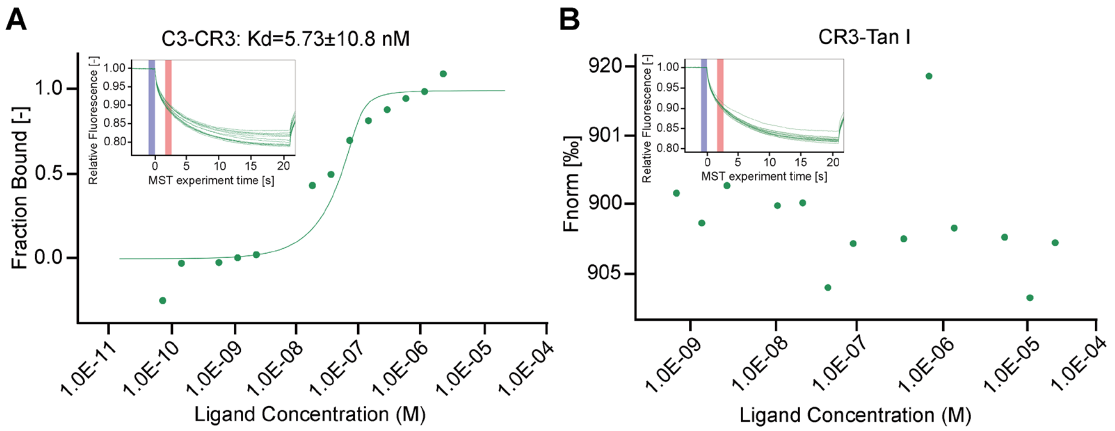

Figure S7. MST was used to analyze the binding of C3 and Tan I to CR3. (A) MST was used to analyze the binding of fluorescently labeled C3 to CR3. **(B)** MST results show no interaction between CR3 and Tan I.

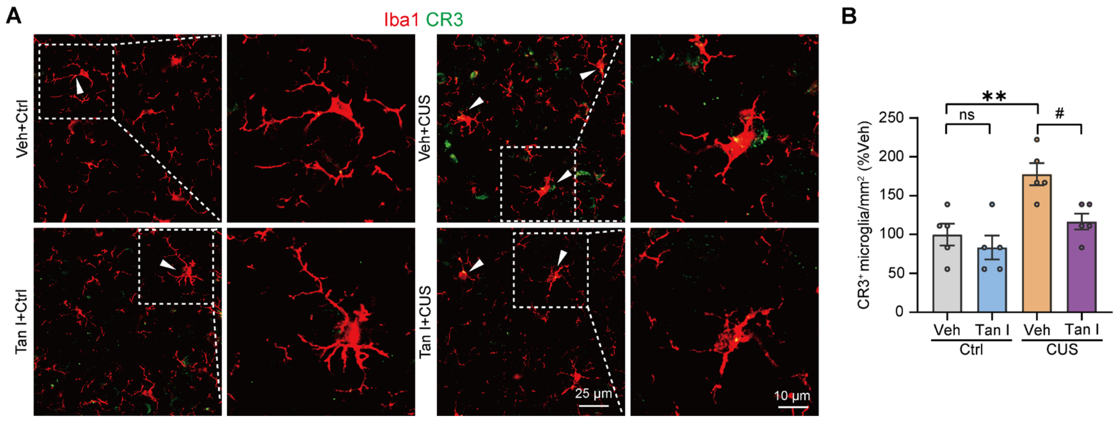

Figure S8. Immunofluorescence analysis of colocalization of CR3 and Iba1 in the mPFC of different groups of mice. (A) Representative images of Iba1 (red) and CR3 (green) in different groups of mice. Scale bar = 25 μm. Zoom in images (bar = 10 μm). **(B)** The quantification of the number of $CR3^+$ microglia in different groups of mice (n = 5). $^{**}P < 0.01$ versus vehicle control group; $^{\#}P < 0.05$ versus vehicle CUS group; ns, not significant. One-way ANOVA with the Tukey's post hoc test. All data are expressed as mean ± SEM.

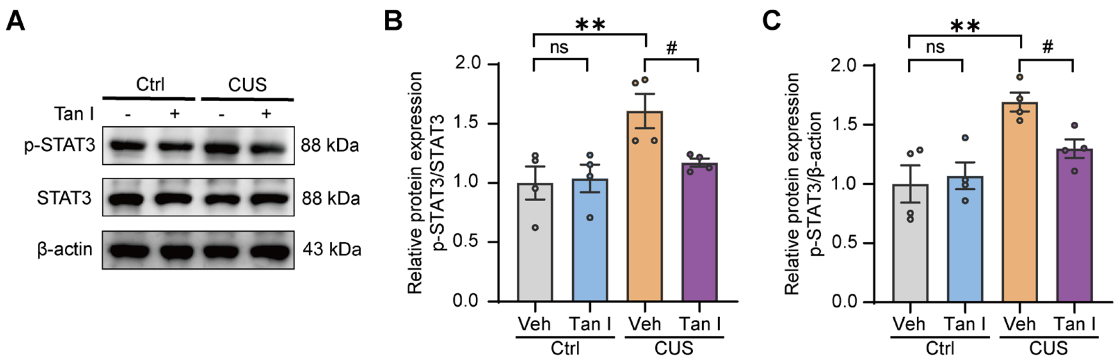

Figure S9. Tan I inhibits STAT3 signaling of CUS mice. (A) The levels of p-STAT3 and STAT3 in the mPFC of different groups of mice were analyzed. **(B-C)** Relative quantification of p-STAT3/STAT3 **(B)** and p-STAT3/β-action **(C)** protein levels in different groups of mice (n = 4). $^{**}P < 0.01$ versus vehicle control group; $^{\#}P < 0.05$ versus vehicle CUS group; ns, not significant. One-way ANOVA with the Tukey's post hoc test. All data are expressed as mean ± SEM.

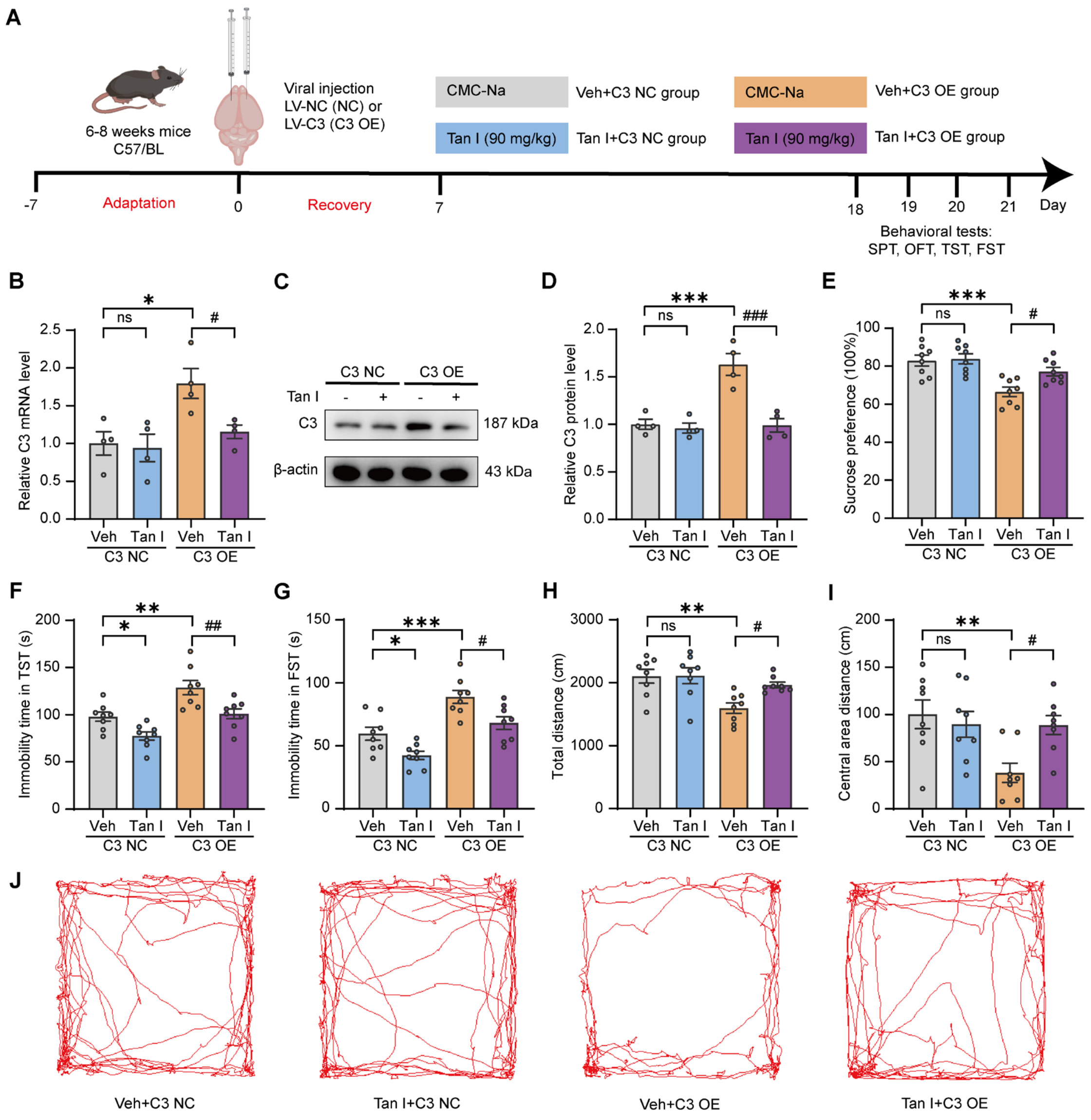

Figure S10. Tan I improves depression-like behavior in C3 OE mice. **(A)** LV-C3 was injected bilaterally into the PFC of WT mice to mimic the pathology of elevated C3. **(B)** The mRNA levels of C3 in the mPFC of C3 OE mice after Tan I treatment were examined (n = 4). **(C)** The levels of C3 in the mPFC were detected. **(D)** Relative quantification of C3 protein levels of C3 OE mice after Tan I treatment (n = 4). **(E)** The mice administered Tan I showed a significant increase in sucrose preference compared to the C3 OE mice (n = 8). **(F)** The mice administered Tan I showed a significant reduction in TST immobilization time compared to the C3 OE mice (n = 8). **(G)** The mice administered Tan I showed a significant reduction in FST immobilization time compared to the C3 OE mice (n = 8). **(H-I)** The mice administered Tan I showed a significant increase in total distance **(H)** and distance in the center **(I)** in the OFT compared with the C3 OE mice (n = 8). **(J)** Representative traces of C3 OE mice movement in the OFT. *$P < 0.05$, **$P < 0.01$, ***$P < 0.001$

versus vehicle C3 NC group; $^{\#}P < 0.05$, $^{\#\#}P < 0.01$, $^{\#\#\#}P < 0.001$ versus vehicle C3 OE group; ns, not significant. One-way ANOVA with the Tukey's post hoc test. All data are expressed as mean ± SEM.

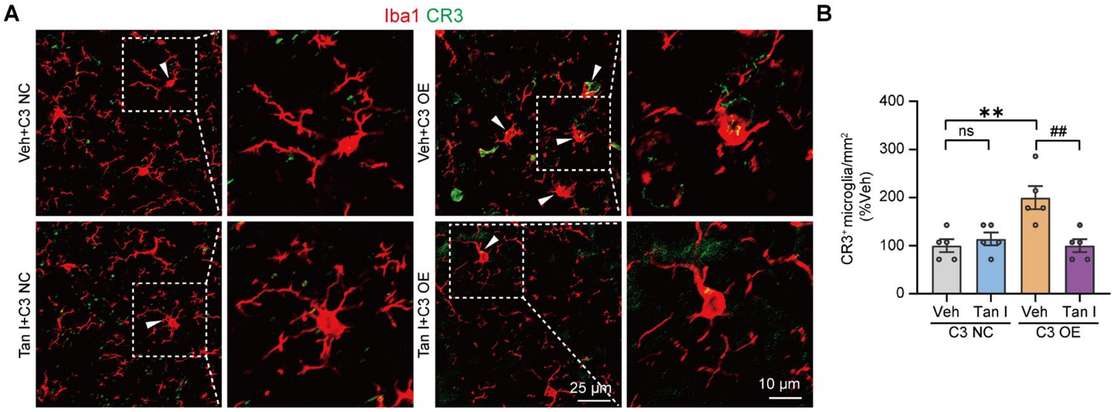

Figure S11. Immunofluorescence analysis of co-localization of CR3 and Iba1 in the mPFC of different groups of mice. (A) Representative images of Iba1 (red) and CR3 (green) in different groups of mice. Zoom in images (bar = 10 μm). Scale bar = 25 μm. **(B)** The quantification of the number of $CR3^+$ microglia in different groups of mice (n = 5). $^{**}P < 0.01$ versus vehicle C3 NC group; $^{\#\#}P < 0.01$ versus vehicle C3 OE group; ns, not significant. One-way ANOVA with the Tukey's post hoc test. All data are expressed as mean ± SEM.

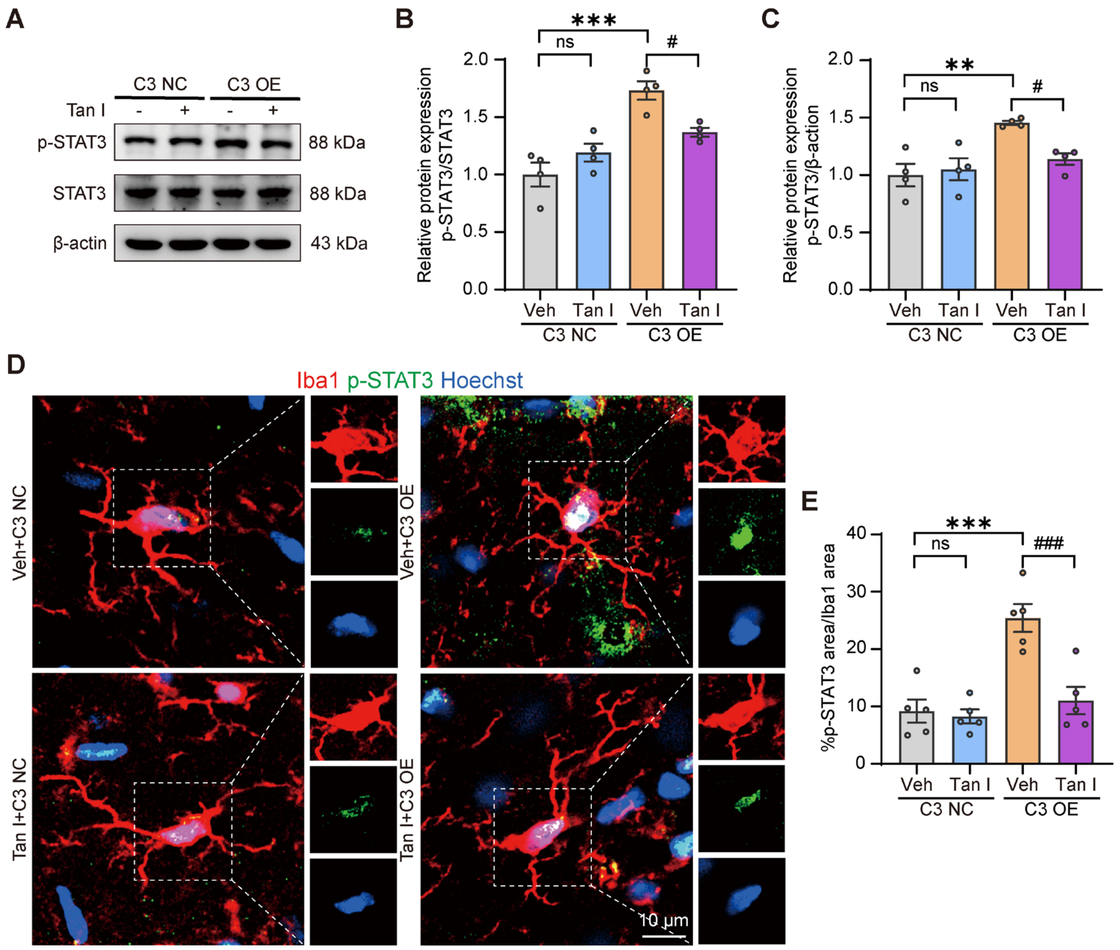

Figure S12. Tan I inhibits STAT3 signaling of C3 OE mice. (A) The levels of p-STAT3 and STAT3 in the mPFC of different groups of mice were analyzed. **(B-C)** Relative quantification of p-STAT3/STAT3 **(B)** and p-STAT3/β-action **(C)** protein levels in different groups of mice (n = 4). **(D)** Representative images of Iba1 (red) and p-STAT3 (green) in different groups of mice. Scale bar = 25 μm. **(E)** The quantification of the ratio of the p-STAT3 area to the Iba1 area in different groups of mice (n = 5). $^{**}P < 0.01$, $^{***}P < 0.001$ versus vehicle C3 NC group; $^{\#}P < 0.05$, $^{\#\#\#}P < 0.001$ versus vehicle C3 OE group; ns, not significant. One-way ANOVA with the Tukey's post hoc test. All data are expressed as mean ± SEM.

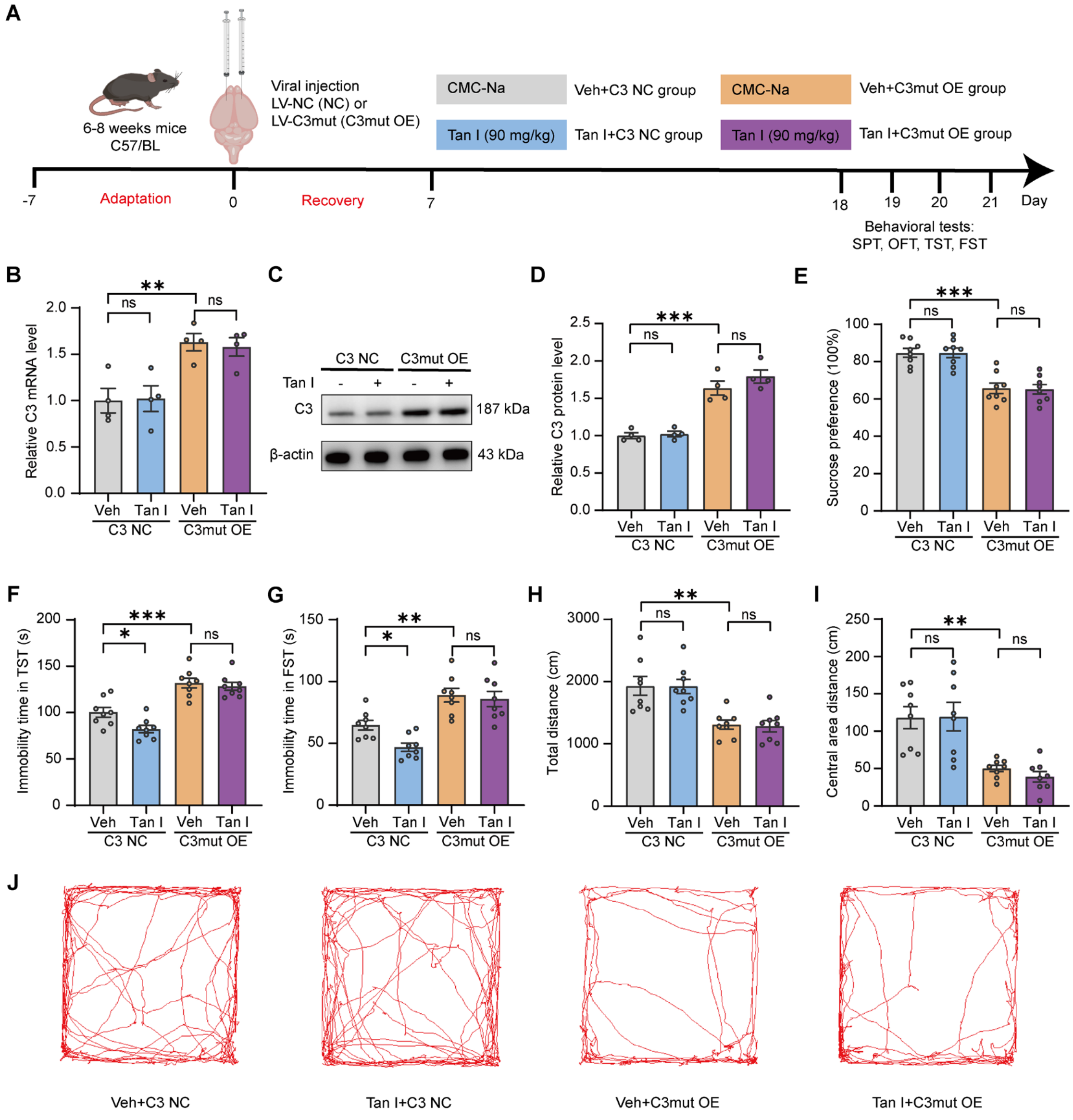

Figure S13. Tan I does not improve depressive-like behavior in C3mut OE mice. **(A)** LV-C3mut was injected bilaterally into the PFC of WT mice. **(B)** The mRNA levels of C3 in the mPFC of C3mut OE mice after Tan I treatment were examined (n = 4). **(C)** The levels of C3 in the mPFC were detected. **(D)** Relative quantification of C3 protein levels of C3mut OE mice after Tan I treatment (n = 4). **(E)** There was no significant increase in sucrose preference in mice administered Tan I compared to C3mut OE mice (n = 8). **(F)** There was no significant reduction in TST immobilization time in mice administered Tan I compared to C3mut OE mice (n = 8). **(G)** There was no significant reduction in FST immobilization time in mice administered Tan I compared to C3mut OE mice (n = 8). **(H-I)** There was no significant increase in total distance **(H)** and distance in the center **(I)** of OFT in mice administered Tan I compared to C3mut OE mice (n = 8). **(J)** Representative traces of C3mut OE mice movement in the OFT. $^{*}P < 0.05$, $^{**}P < 0.01$, $^{***}P < 0.001$

versus vehicle C3 NC group; ns, not significant. One-way ANOVA with the Tukey's post hoc test. All data are expressed as mean ± SEM.

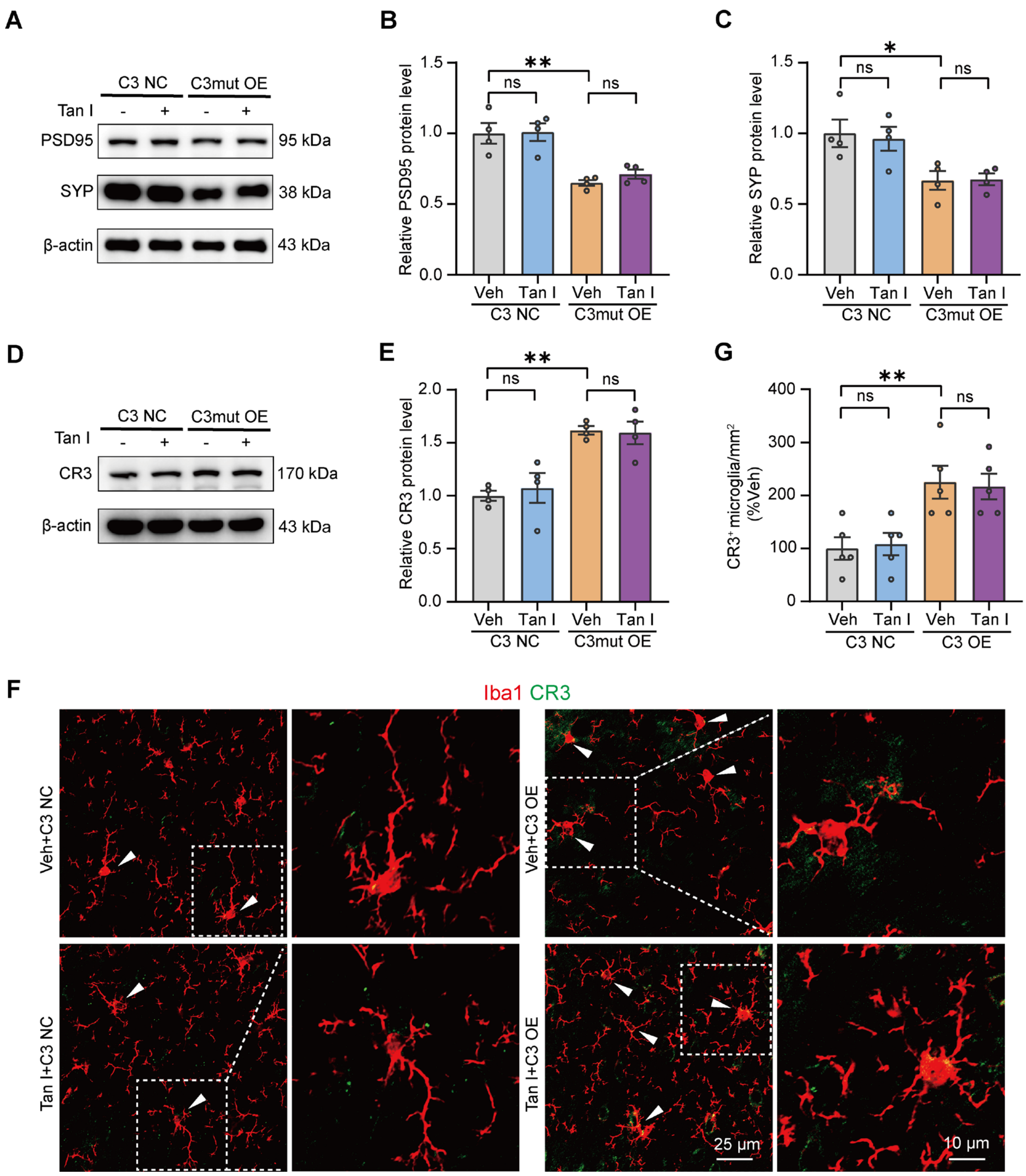

Figure S14. Tan I do not inhibit microglia-mediated synaptic engulfment in C3mut OE mice. **(A)** The levels of PSD95 and SYP in the mPFC were analyzed. **(B-C)** Relative quantification of PSD95 and SYP protein levels of C3mut OE mice after Tan I treatment (n = 4). **(D)** The levels of CR3 in the mPFC were analyzed. **(E)** Relative quantification of CR3 protein levels of C3mut OE mice after Tan I treatment (n = 4). **(F)** Representative images of Iba1 (red) and CR3 (green) in the mPFC. Scale bar = 25 μm. Zoom in images (bar = 10 μm). **(G)** The quantification of the number of $CR3^+$ microglia in different groups of mice (n = 5). $^*P < 0.05$, $^{**}P < 0.01$ versus vehicle C3 NC group; ns, not significant. One-way ANOVA with the Tukey's post hoc test. All data are expressed as mean ± SEM.

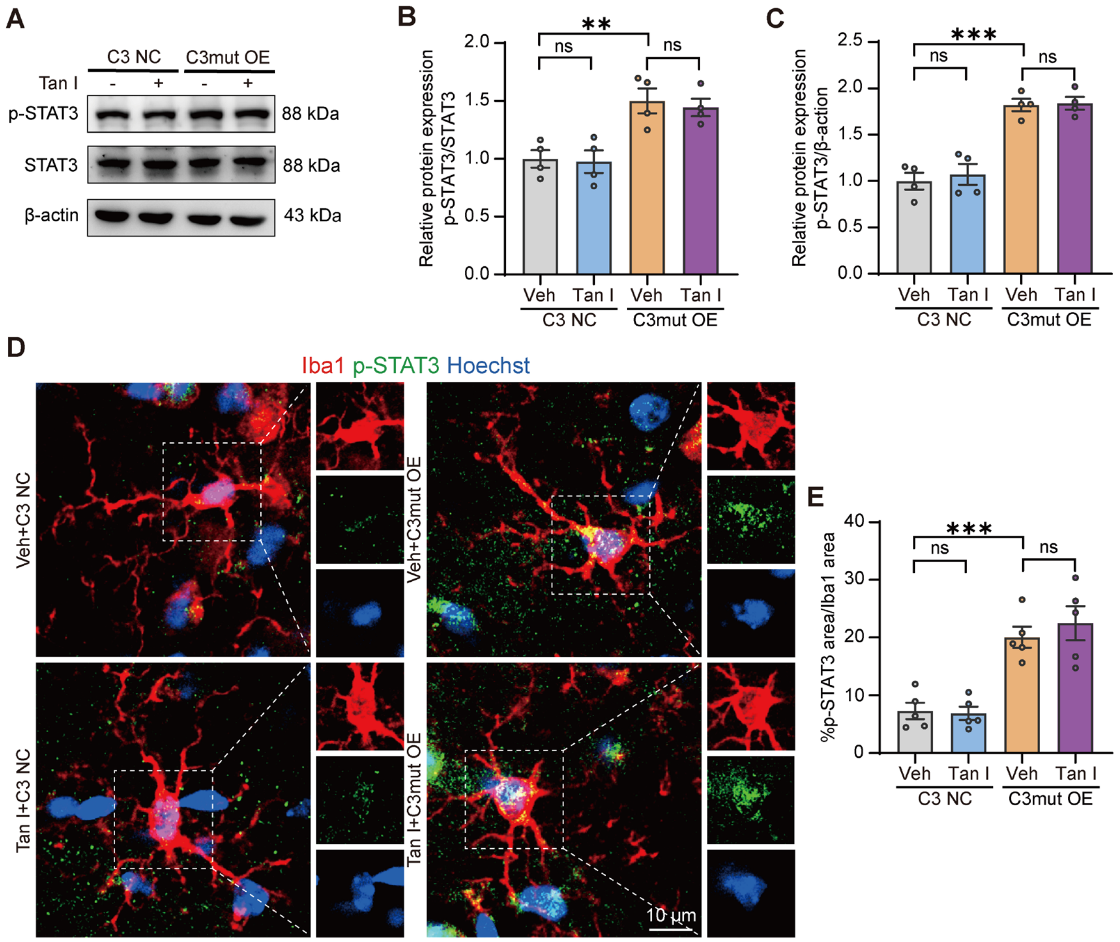

Figure S15. Tan I does not inhibit STAT3 signaling of C3mut OE mice. (A) The levels of p-STAT3 and STAT3 in the mPFC of different groups of mice were analyzed. **(B-C)** Relative quantification of p-STAT3/STAT3 **(B)** and p-STAT3/β-action **(C)** protein levels in different groups of mice (n = 4). **(D)** Representative images of Iba1 (red) and p-STAT3 (green) in different groups of mice. Scale bar = 25 μm. **(E)** The quantification of the ratio of the p-STAT3 area to the Iba1 area in different groups of mice (n = 5). $^{**}P < 0.01$, $^{***}P < 0.001$ versus vehicle C3 NC group; ns, not significant. One-way ANOVA with the Tukey's post hoc test. All data are expressed as mean ± SEM.

Supplementary tables

Table 1 S1. Drugs, antibodies, chemicals, and instruments used in this study

Reagent or resource	Source
**Drug**	
Tanshinone I (B064161, HPLC > 98%)	Beijing konoscience Technology Co.
Fluoxetine (HY-B0102, HPLC > 98%)	MCE
**Protein**	
C3 (PR02855, HPLC > 95%)	ABclonal
CR3 (Ag16327, HPLC > 95%)	Proteintech
C1q (URPD207Hu01, HPLC > 95%)	Cloud-Clone
C4 (URPA888Hu01, HPLC > 95%)	Cloud-Clone
**Antibodies**	
c-Fos (sc-166940, 1:200)	Santa Cruz
PSD95 (sc-32290, 1:200 or 1:500)	Santa Cruz
SYP (17785-1-AP, 1:200 or 1:2000)	Proteintech
CaMKIIα (13730-1-AP, 1:100)	Proteintech
GAD67 (10408-1-AP, 1:100)	Proteintech
C3 (21337-1-AP, 1:200)	Proteintech
C3 (sc-28294, 1:500)	Santa Cruz
CR3 (FITC-65055, 1:200 or 1:1000)	Proteintech
Iba-1 (ab289874, 1:100)	Abcam
Iba-1 (MA5-27726,1:200)	Thermo Fisher
CD68 (28058-1-AP, 1:100)	Proteintech
NeuN (26975-1-AP, 1:100)	Proteintech
COX-2 (27308-1-AP, 1:500)	Proteintech
iNOS (ab15323, 1:250)	Abcam
STAT3 (10253-2-AP, 1:2000)	Proteintech
p-STAT3 (ET1607-39, 1:1000)	HUABIO
β-actin (AC038, 1:10,000)	ABclonal

Alexa 488-conjugated donkey anti-mouse IgG (A32766, 1:500)	Invitrogen
Alexa 488-conjugated donkey anti-rabbit IgG (A21206, 1:500)	Invitrogen
Alexa 488-conjugated donkey anti-goat IgG (A11055, 1:500)	Invitrogen
Alexa 546-conjugated donkey anti-mouse (A10036, 1:500)	Invitrogen
Alexa 546-conjugated donkey anti-rabbit IgG (A10040, 1:500)	Invitrogen
Alexa 647-conjugated donkey anti-mouse IgG (A31571, 1:500)	Invitrogen
Alexa 647-conjugated donkey anti-rabbit IgG (A31573, 1:500)	Invitrogen
Hochest 33342 (1: 1000)	Heychem
Horseradish peroxidase-polyclonal sheep Anti-Mouse IgG (5220-0341, 1:5000)	SeraCare,
Horseradish peroxidase-conjugated sheep Anti-Rabbit IgG. (5220-0336, 1:5000)	SeraCare
**Other Materials**	
BV2 cells (mouse microglial cells)	Procell Life
CMC-Na (9004-32-4)	Sinopharm Chemical Reagent Co., LTD
C3 Lentiviral (LV) and Control LV	OBiO Technology
Dulbecco's Modified Eagle Medium (DMEM) (FI101-01)	TransGen Biotech
Fetal bovine serum (FBS) (A5670701)	Gibco
IL-1β ELISA kit (F2132-B)	Shanghai Kexing Trading Co., Ltd
TNF-α ELISA kit (F2040-B)	Shanghai Kexing Trading Co., Ltd
IL-10 ELISA kit (F2176-A)	Shanghai Kexing Trading Co., Ltd
TGF-β ELISA kit (F2686-A)	Shanghai Kexing Trading Co., Ltd

MDA assay kit (S0131S)	Beyotime
BCA protein assay kit (P0011)	Beyotime
CCK-8 Cell Proliferation and Cytotoxicity Assay Kit (CA1210)	Solarbio
TRIzol Reagent (15596018)	Thermofisher
SuperScript™ II Reverse Transcriptase (1896649)	Invitrogen
RNA 6000 Nano LabChip Kit (5067-1511)	Agilent
Hematoxylin dye and eosin dye (G1005-1 G1005-2)	Servicebio
Pronase (10165921001)	Roche
**Instrument**	
Pharmascan 70/16 MRI system	BRUKER
Transmission electron microscopy (JEM-1200EX)	JEOL
AB SCIEX 4500 QTRAP	SCIEX
UltiMate 3000 UPLC	Shimadzu
Digital slice scanner panoramic multilayer	Servicebio
Illumina Novaseq™ 6000	LC-Bio Technology CO., Ltd.
7500 Real-time PCR system	Applied Biosystems
Cytation C10	BioTek
Monolith NT.115 instrument	Nano-Temper Technologies
Q Exactive HF-X	Thermo Fisher

Table S2. Primers used for real-time PCR

Gene name	Primer sequence (5′-3′)	Orientation
C3	CCCTGGCCCTGATGAACAAA	Forward
	TTGAGCCAGCGCACTACAGG	Reverse
C1qa	GGCAAACCTGGCAATGTGG	Forward
	CCAAGCGTCATTGGGTTCTG	Reverse
C1qb	CAACCAGGCACTCCAGGGATAA	Forward
	CCAACTTTGCCTGGAGTCCCAG	Reverse
Cx3cr1	GTTCCAAAGGCCACAATGTC	Forward
	TGAGTGACTGGCACTTCCTG	Reverse
GAPDH	GAAGGTCGGTGTGAACGGATTTGGC	Forward
	GATGGGCTTCCCGTTGATGACAAGC	Reverse
